# Proteasome dysfunction in alveolar type 2 epithelial cells is associated with acute respiratory distress syndrome

**DOI:** 10.1038/s41598-019-49020-4

**Published:** 2019-08-29

**Authors:** Sneha Sitaraman, Cheng-Lun Na, Li Yang, Alyssa Filuta, James P. Bridges, Timothy E. Weaver

**Affiliations:** 1 Division of Pulmonary Biology, Cincinnati Children’s Hospital Medical Center, Cincinnati, Ohio 45229 USA; 2 Division of Pulmonary, Critical Care and Sleep Medicine, National Jewish Health, Denver, Colorado 80206 USA

**Keywords:** Proteasome, Respiratory distress syndrome

## Abstract

Proteasomes are a critical component of quality control that regulate turnover of short-lived, unfolded, and misfolded proteins. Proteasome activity has been therapeutically targeted and considered as a treatment option for several chronic lung disorders including pulmonary fibrosis. Although pharmacologic inhibition of proteasome activity effectively prevents the transformation of fibroblasts to myofibroblasts, the effect on alveolar type 2 (AT2) epithelial cells is not clear. To address this knowledge gap, we generated a genetic model in which a proteasome subunit, RPT3, which promotes assembly of active 26S proteasome, was conditionally deleted in AT2 cells of mice. Partial deletion of RPT3 resulted in 26S proteasome dysfunction, leading to augmented cell stress and cell death. Acute loss of AT2 cells resulted in depletion of alveolar surfactant, disruption of the alveolar epithelial barrier and, ultimately, lethal acute respiratory distress syndrome (ARDS). This study underscores importance of proteasome function in maintenance of AT2 cell homeostasis and supports the need to further investigate the role of proteasome dysfunction in ARDS pathogenesis.

## Introduction

Cellular quality control (QC) is governed by two distinct but interconnected processes, the ubiquitin-proteasome system and the autophagy-lysosome system^1^. Macroautophagy, the major autophagic pathway, regulates the turnover of bulk aggregates and damaged organelles whereas proteasomes regulate the turnover of soluble unfolded, misfolded, short-lived, and regulatory proteins that control various cellular functions, such as cell cycle progression^2^. Cellular QC is an attractive therapeutic target because of the ease of pharmacological manipulation and the increasing availability of inhibitors and activators. Bortezomib, a reversible inhibitor of proteasome activity, is in clinical use for treatment of multiple myeloma and mantle cell lymphoma^3^, and is reported to have anti-fibrotic effects in multiple animal models [reviewed in^4^]. Mutlu *et al*. reported that Bortezomib treatment in mice largely prevented the fibrotic response associated with bleomycin-induced lung injury^5^; however, in two other studies of bleomycin-treated mice, Bortezomib^6^ or Oprozomib^7^, an irreversible proteasome inhibitor, did not diminish the fibrotic response. Interestingly, all three studies confirmed that inhibition of proteasome activity in TGF-B-stimulated fibroblasts prevented differentiation to myofibroblasts *in vitro*. However, translation of these anti-fibrotic effects of proteasome inhibitors to the disease setting may be influenced by differential cellular responses to proteasome inhibition *in vivo*. The therapeutic use of proteasome inhibitors is further complicated by reports that some patients treated with Bortezomib developed significant pulmonary complications including acute respiratory distress syndrome (ARDS)^8–12^. These data underscore the need to further investigate the use of proteasome inhibitors in chronic lung diseases, including identification of potential cell-specific responses to proteasome inhibition.

RPT3, also known as TBP7 or *Psmc4*, is an AAA^+^-ATPase subunit of the 19S regulatory particle of the 26S proteasome^13^. The 19S regulatory particle, which caps the 20S catalytic core on one or both ends, is involved in recognition, unfolding and delivery of ubiquitinated substrates to the 20S core for degradation. In addition to hydrolysis of ATP, the six-member ATPase family (RPT1-6) is required for the precise assembly of the 26S proteasome and gating of the 20S core complex^14–16^. Several proteasome subunits, including RPT3, were shown to be dysregulated in degenerative diseases such as Alzheimer’s and Parkinson’s disease^17–19^. Deletion of RPT3 from motor neurons in mice resulted in chronic neurodegeneration with formation of aggregates and inclusions, similar to the histopathology observed in patients with amyotrophic lateral sclerosis^20^. Muscle-specific deletion of RPT3 resulted in disorganized skeletal muscle fibers, loss of muscle mass and decreased ambulation^21^. Global deletion of RPT3 resulted in early embryonic lethality due to defective blastocyst development, emphasizing the requirement of RPT3 for cell survival^22^.

Consistent with the degenerative phenotypes observed in RPT3-deficient mice, we hypothesized that RPT3 deficiency in alveolar type 2 (AT2) cells would predispose to fibrosis, i.e. proteasome dysfunction would result in formation and accumulation of aggregates, resulting in chronic AT2 cell injury and a persistent fibroproliferative response. Unexpectedly, partial deletion of RPT3 resulted in lethality following sudden onset of respiratory distress syndrome. Alveolar epithelial barrier structure and function were disrupted in RPT3^AT2Δ/Δ^ mice highlighting the importance of proteasome function in maintenance of alveolar homeostasis. These findings suggest that the risk of respiratory failure is a concern for some patients undergoing high dose and/or extended proteasome inhibitor therapy.

## Results

### Partial deletion of RPT3 in AT2 cells results in lethality

**Figure 1:**
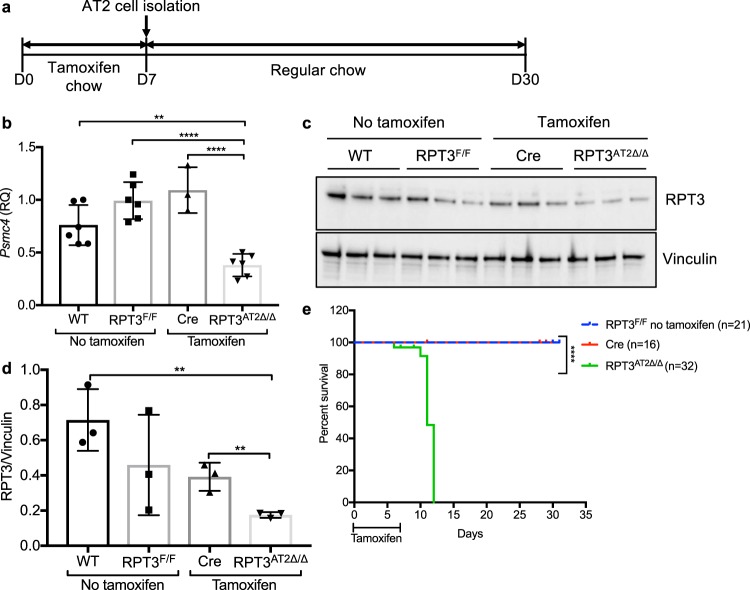
Targeted deletion of RPT3 in AT2 cells is lethal. (**a**) *Sftpc*^WT/CreER^:RPT3^F/F^ (RPT3^F/F^) and *Sftpc*^WT/CreER^ (Cre) mice were fed tamoxifen chow for 7 days to promote recombination at the *Psmc4* (RPT3) locus in AT2 cells, then switched to regular chow on day 7. The efficiency of recombination was assessed in AT2 cells (isolated on day 7) by quantitative PCR (**b**) and Western blotting (**c**). (**d**) Densitometry of Western blot in c. **p < 0.01, ****p < 0.0001 by one-way ANOVA with Tukey’s multiple comparison test. RQ: relative quantitation. (**e**). Kaplan Meier survival curve. ****p < 0.0001 by Mantel-Cox test. Full-length blots are presented in Supplementary Fig. 9.

RPT3 was conditionally deleted from AT2 cells by crossing RPT3^F/F^ mice with *Sftpc*-CreER^T2^ driver mice (hereafter referred to as Cre mice) and feeding 8-12-week old female and male *Sftpc*^WT/CreER^:RPT3^F/F^ mice tamoxifen chow for 7 days. Quantitative PCR and Western blot analyses of AT2 cells isolated from RPT3^AT2Δ/Δ^ mice demonstrated a reduction of RPT3 mRNA (*Psmc4*) by 50% (WT = 0.76 ± 0.19, *Sftpc*^WT/CreER^:RPT3^F/F^ = 0.992 ± 0.18, *Sftpc*^WT/CreER^ = 1.092 ± 0.2, RPT3^AT2Δ/Δ^ = 0.38 ± 0.11) and protein by 75% (WT = 0.72 ± 0.18, *Sftpc*^WT/CreER^:RPT3^F/F^ = 0.46 ± 0.3, *Sftpc*^WT/CreER^ = 0.4 ± 0.1, RPT3^AT2Δ/Δ^ = 0.18 ± 0.02) compared to WT controls after 7 days of tamoxifen chow (Fig. 1a–d). In the absence of tamoxifen, RPT3 mRNA and protein levels in *Sftpc*^WT/CreER^:RPT3^F/F^ (RPT3^F/F^) mice were not significantly different from WT mice, suggesting little or no spontaneous Cre-mediated recombination at the *Psmc4* locus (Fig. 1b–d). Additionally, a primer probe set directed to exons 3–4, upstream of the targeted region (exons 7–11)^20^, demonstrated no alterations in RPT3 mRNA levels, indicating that Cre-mediated excision resulted in the formation of a stable, but truncated mRNA (Supplementary Fig. S1b).

AT2 cell-specific deletion of RPT3 resulted in acute morbidity and lethality (Fig. 1e). Aversion to tamoxifen chow was observed in both RPT3^AT2Δ/Δ^ and Cre mice: both RPT3^AT2Δ/Δ^ and Cre mice lost up to 12% of body weight after 4 days of treatment but began to gain weight after day 5 (Supplementary Fig. S1a). Daily assessment of health indicated that RPT3^AT2Δ/Δ^ and Cre mice gained weight after transition to regular chow and were active. However, on day 10, RPT3^AT2Δ/Δ^ mice began to lose weight (Supplementary Fig. S1a). On day 11, all RPT3^AT2Δ/Δ^ mice experienced a precipitous decline in health and activity: 53% of RPT3^AT2Δ/Δ^ mice (n = 17/32) lost 7.5% of body weight from day 10 (12% loss from day 0) leading to morbidity and death within 3–4 hours, and 47% of RPT3^AT2Δ/Δ^ mice (n = 15/32) lost greater than 20% body weight and were immediately euthanized. In contrast to RPT3^AT2Δ/Δ^ mice, Cre mice maintained on tamoxifen chow for 7 days were active and healthy on day 11 and continued to gain weight (Supplementary Fig. S1a).

To identify a deletion strategy that did not result in acute morbidity, mice were treated with tamoxifen for shorter periods of time. Mice were fed tamoxifen chow for 1, 3, 4 or 5 days, transitioned to regular diet, monitored daily, and surviving mice euthanized 3.5 weeks after removal of tamoxifen chow (Supplementary Fig. S1c). Tamoxifen treatment for 5 days resulted in lethality on day 11 of the study (n = 2/3), similar to the 7-day tamoxifen treatment regimen; therefore, recombination efficiency at the *Psmc4* locus was assessed following a 4-day treatment regimen. Quantitative PCR and Western blot analyses of isolated AT2 cells detected no significant changes in *Psmc4* mRNA or RPT3 protein and all mice survived to day 35 when the experiment was terminated (Supplementary Fig. S1e,f). Since recombination was minimal after 4 days of tamoxifen treatment, all further studies were conducted using the 7-day tamoxifen treatment regimen.

### RPT3 deletion is associated with acute loss of AT2 cells

**Figure 2:**
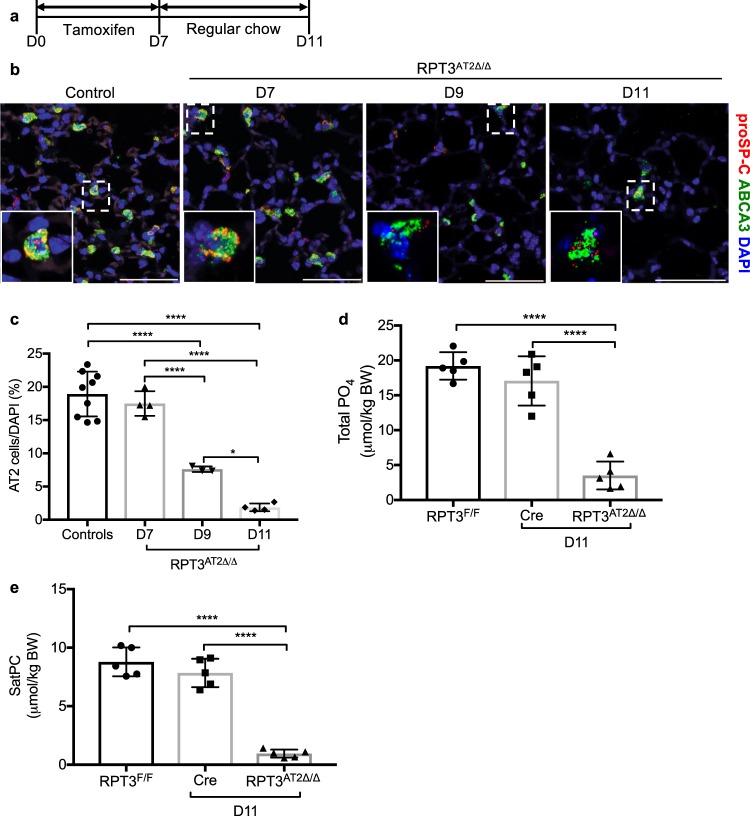
Targeted deletion of RPT3 in AT2 cells leads to AT2 cell loss. (**a**) *Sftpc*^WT/CreER^:RPT3^F/F^ and *Sftpc*^WT/CreER^ (Cre) mice were fed tamoxifen chow for 7 days, then switched to regular chow on day 7. (**b**) Representative maximum intensity projections of confocal z-stacks of lung sections stained for proSP-C and ABCA3. Insets show proSP-C^+^ ABCA3^+^ cell. Scale bars = 50 μm. (**c**) Frequency of AT2 cells assessed by morphometric analysis of b. Controls included both RPT3^F/F^ mice (*Sftpc*^WT/CreER^:RPT3^F/F^ mice without tamoxifen treatment) and Cre mice that were fed tamoxifen chow for 7 days. DAPI counts remained unchanged across all groups (not shown). (**d**) Concentration of total phosphate (PO_4_) in bronchoalveolar lavage fluid (BALF), normalized to body weight (BW). (**e**) Concentration of saturated phosphatidylcholine (SatPC) in BALF, normalized to BW. Total PO_4_ and SatPC were measured in mice euthanized on day 11. *p < 0.05, ****p < 0.0001 by one-way ANOVA with Tukey’s multiple comparison test.

Given the development of acute respiratory failure, the number of AT2 cells was analyzed in RPT3^AT2Δ/Δ^ mice euthanized in the 5-day period (days 7–11) prior to presentation of respiratory symptoms. Flow cytometric analysis of lung single cell suspensions on day 11 demonstrated a 53.1% decrease in the frequency of CD326^+^ cells in RPT3^AT2Δ/Δ^ mice [*Sftpc*^WT/CreER^:RPT3^F/F^ = 12.8 ± 2.9 (% of live cells), RPT3^AT2Δ/Δ^ = 6 ± 2.2 (% of live cells)], which includes both airway and alveolar epithelial cells (Supplementary Fig. S2a). To specifically assess the loss of AT2 cells, morphometric analyses were performed on lung sections stained with proSP-C and ABCA3 (Fig. 2b), both of which mark mature AT2 cells. No change in AT2 cell frequency was observed after 7 days of tamoxifen treatment (controls = 18.9 ± 3.4, RPT3^AT2Δ/Δ^ = 17.5 ± 1.9) (Fig. 2c). Compared to controls, which included *Sftpc*^WT/CreER^:RPT3^F/F^ mice without tamoxifen and *Sftpc*^WT/CreER^ mice with tamoxifen treatment, RPT3^AT2Δ/Δ^ mice demonstrated a 59.8% (7.6 ± 4.2) and 90.5% (1.8 ± 0.6) reduction in AT2 cell frequency on days 9 and 11, respectively.

AT2 cells synthesize and secrete pulmonary surfactant, which lowers surface tension and prevents alveolar collapse at end expiration. Surfactant is composed primarily of phospholipids with an abundance of phosphatidylcholine (PC); approximately 50% of PC is dipalmitoylphosphatidylcholine (SatPC) which is essential for the surface tension reducing property of surfactant^23^. To determine if respiratory distress in RPT3^AT2Δ/Δ^ mice was associated with deficiency of surfactant phospholipids, alveolar surfactant pool size was assessed on day 11. Prior to presentation of symptoms on day 11, RPT3^AT2Δ/Δ^ mice demonstrated an 81.7% decrease in total phospholipid levels (*Sftpc*^WT/CreER^:RPT3^F/F^ = 19.2 ± 2, *Sftpc*^WT/CreER^ = 17.07 ± 3.5, RPT3^AT2Δ/Δ^ = 3.5 ± 2) and 89.1% decrease in SatPC levels (*Sftpc*^WT/CreER^:RPT3^F/F^ = 8.8 ± 1.2, *Sftpc*^WT/CreER^ = 7.9 ± 1.2, RPT3^AT2Δ/Δ^ = 0.96 ± 0.3) in bronchoalveolar lavage fluid (BALF) (Fig. 2d,e) compared to *Sftpc*^WT/CreER^:RPT3^F/F^ mice, consistent with loss of AT2 cells. Tamoxifen treatment had no effect on surfactant pool size or SatPC levels in Cre mice.

### AT2 cell-specific deletion of RPT3 leads to disruption of the alveolar epithelial barrier

**Figure 3:**
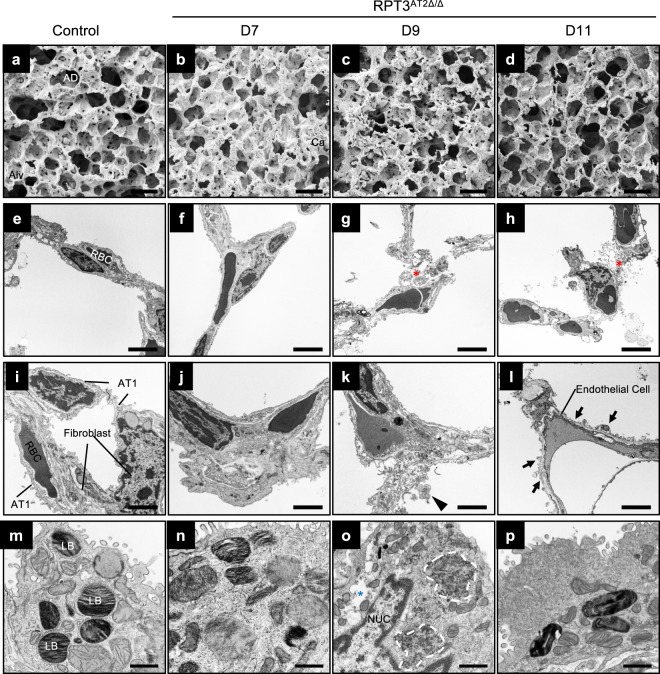
Altered alveolar epithelial structure in RPT3^AT2Δ/Δ^ mice. (**a**–**d**) SEM analyses of alveolar architecture on days 7, 9 and 11 after tamoxifen treatment (days 1–7). AD: alveolar duct, Alv: alveolus and alveolar walls, Ca: capillary. n = 2 mice/genotype. (**e**–**p**) TEM of alveolar walls. Pronounced membrane blebbing (arrows; l) was observed in AT1 cells. Arrowhead (k) points to cellular debris. Dashed circle (**o**): accumulation of electron dense macromolecules. Red asterisks (**g**,**h**): disintegrated alveolar walls. Blue asterisk (**o**): vacuole in AT2 cell. LB: lamellar body, NUC: nucleus. Scale bars = 50 μm (**a**–**d**), 5 μm (**e**–**h**), 2.5 μm (**i**–**l**) and 1 μm (m-p). n = 2–4 mice/genotype. Controls included both *Sftpc*^WT/CreER^:RPT3^F/F^ mice without tamoxifen treatment and Cre mice that were fed tamoxifen chow for 7 days.

RPT3 deficiency resulted in an acute and rapid loss of AT2 cells, followed by sudden onset of respiratory distress and death on day 11. Paradoxically, analysis of H&E stained lung sections of RPT3^AT2Δ/Δ^ mice prior to presentation of symptoms on day 11 revealed normal lung structure with only very mild inflammation (Supplementary Fig. S2b–d). Consistent with histological analyses, alveolar structure appeared relatively unaffected when examined by scanning electron microscopy (SEM) (Fig. 3a–d). However, transmission electron microscopy (TEM) identified cell debris (Fig. 3g,h), damaged alveolar type 1 (AT1) epithelial cells with pronounced membrane blebbing (Fig. 3k–l) and alveoli with vacuolated AT2 cells (Fig. 3o). Additionally, AT2 cells from RPT3^AT2Δ/Δ^ mice contained aggregates (Fig. 3o) and electron dense lamellar bodies (Fig. 3p) compared to control AT2 cells (*Sftpc*^WT/CreER^:RPT3^F/F^ mice without tamoxifen and *Sftpc*^WT/CreER^ mice with tamoxifen treatment). Consistent with TEM analyses, lung sections stained with T1α (Podoplanin) revealed damaged AT1 cells on day 11, including cells that appeared distended, with T1α^+^ debris in the airspaces (Supplementary Fig. S3a). Importantly, ultrastructural changes in AT1 cells were detected as early as day 9 (Fig. 3k) coincident with a 60% decrease in the frequency of AT2 cells (Fig. 2c). Structural changes were more pronounced in the alveolar epithelium compared to the endothelium; significant qualitative changes were not observed in endothelial cells by TEM analyses (Fig. 3l) or by confocal imaging of lung sections stained with EMCN (Endomucin) (Supplementary Fig. S3b). Collectively, these data indicate that RPT3 deficiency leads to AT2 cell injury and disruption of the alveolar epithelial barrier.

To assess the impact of epithelial damage on barrier function, epithelial barrier permeability was measured on day 11, prior to presentation of any symptoms. Mice were intravenously injected with albumin conjugated to FITC, followed by measurement of FITC fluorescence in BALF. RPT3^AT2Δ/Δ^ mice demonstrated a 3.5-fold increase in FITC fluorescence compared to *Sftpc*^WT/CreER^:RPT3^F/F^ mice (*Sftpc*^WT/CreER^:RPT3^F/F^ = 0.23 ± 0.1, *Sftpc*^WT/CreER^ = 0.23 ± 0.1, RPT3^AT2Δ/Δ^ = 0.8 ± 0.4), indicating an increase in barrier permeability in response to RPT3 deficiency (Supplementary Fig. S4a). Consistent with increased barrier permeability, a 4.4-fold increase in protein concentration was observed in BALF recovered from an independent group of RPT3^AT2Δ/Δ^ mice compared to *Sftpc*^WT/CreER^:RPT3^F/F^ mice (*Sftpc*^WT/CreER^:RPT3^F/F^ = 0.5 ± 0.1, *Sftpc*^WT/CreER^ = 0.74 ± 0.4, RPT3^AT2Δ/Δ^ = 2.2 ± 0.96) (Supplementary Fig. S4b). Although the latter group of RPT3^AT2Δ/Δ^ mice demonstrated a significant increase in neutrophil frequency in BALF (Supplementary Fig. S4c), the total number of immune cells remained unchanged (Supplementary Fig. S4d), consistent with the mild inflammation observed in histological analyses (Supplementary Fig. S2d’). Collectively, these data confirm that AT2 cell-specific deletion of RPT3 leads to altered alveolar epithelial barrier structure and function.

### Adaptive changes in response to RPT3 deficiency

To identify transcriptomic changes in response to partial deletion of RPT3, RNA sequencing was performed on AT2 cells isolated from RPT3^AT2Δ/Δ^ and Cre mice on day 9 after tamoxifen treatment (days 1–7); day 9 was chosen to identify gene expression changes associated with a 60% decrease in AT2 cell frequency (Fig. 2c). In response to RPT3 deficiency, 6569 genes were differentially expressed, with 53.4% genes (n = 3509/6569) upregulated and 46.6% genes (3060/6569) downregulated (Supplementary Fig. S5a). Gene ontology (GO) analysis revealed significant enrichment of genes associated with catabolic and metabolic processes, ubiquitin-dependent proteolysis, cell stress, cell death and apoptosis (Supplementary Table S1). Differential expression of 175 proteasome-associated genes was observed in response to RPT3 deficiency (Supplementary Fig. S5b), with 83.1% genes (n = 148/178) upregulated and 16.9% genes (n = 30/178) downregulated. Upregulated genes included majority of the proteasome subunits and regulators including *Psmc4* (RPT3) (Supplementary Table S2 and Fig. S5c,d). Analysis of RNA sequencing track data and qPCR on isolated AT2 cells using primer-probe sets directed to exons 3–4 and 9–11 revealed that increase in *Psmc4* expression was due to increased formation of a stable, truncated mRNA (Supplementary Figs. S1b and S5e). Accordingly, Western blot analyses of isolated AT2 cells revealed a decrease in RPT3 protein on day 9 by 73% (*Sftpc*^WT/CreER^:RPT3^F/F^ = 0.194 ± 0.011, RPT3^AT2Δ/Δ^ = 0.053 ± 0.012) (Supplementary Fig. S5f,g).

### RPT3 deletion leads to accumulation of proteasome substrates in AT2 cells

**Figure 4:**
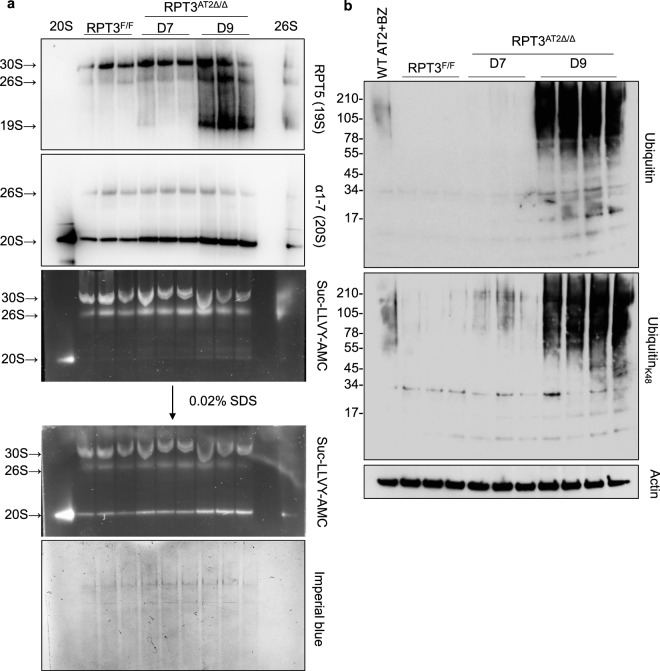
RPT3 deficiency is associated with increased 20S protease activity but decreased degradation of ubiquitinated substrates. (**a**) 30 μg of AT2 cell lysates were resolved by native gel electrophoresis followed by immunoblotting for 20S α subunits (1, 2, 3, 5, 6, 7) and 19S subunit RPT5, and overlay assay with Suc-LLVY-AMC substrate. 0.5 μg of human 26S proteasome and bovine 20S proteasome were used as controls. Addition of SDS stimulates 20S peptidase activity. Gel was stained post transfer with imperial blue to assess protein transfer. (**b**) 20 μg of AT2 cell lysates were separated by SDS-PAGE followed by immunoblotting for polyubiquitin and K-48 linked polyubiquitin. WT AT2 cells cultured on 100% Cultrex were treated with 10 nM Bortezomib (BZ) for 48 hours. Cultrex was dissolved using Cell Recovery Solution and cells were collected in 2X sample buffer containing BME for Western blotting. RPT3^F/F^ - *Sftpc*^WT/CreER^:RPT3^F/F^ mice without tamoxifen treatment. Full-length blot for Actin is presented in Supplementary Fig. 9.

The 26S proteasome comprises a 20S catalytic core and a 19S regulatory particle which caps one or both ends of the 20S complex. The 19S complex recognizes ubiquitinated substrates targeted for degradation by the 20S core. RPT3 is an AAA^+^-ATPase subunit of the 19S complex and is required for assembly of the 26S proteasome. Native gel electrophoresis of isolated AT2 cells was performed to determine the effect of increased expression of genes encoding both 19S and 20S proteasome subunits (Supplementary Table S2). Immunoblotting of native gels demonstrated an increase in 20S α1-7 subunits and the 19S subunit RPT5 (Fig. 4a), consistent with differential gene expression analyses (Supplementary Fig. S5c). Accumulation of 20S proteasome content was accompanied by an increase in 20S chymotrypsin-like activity in AT2 cells isolated from RPT3^AT2Δ/Δ^ mice on day 9 compared to *Sftpc*^WT/CreER^:RPT3^F/F^ mice (Fig. 4a). However, SDS-PAGE of AT2 cells isolated from RPT3^AT2Δ/Δ^ mice revealed a dramatic accumulation of polyubiquitinated and K48-linked polyubiquitinated substrates (Fig. 4b). Collectively, these data indicate that although RPT3 deficiency is associated with an increase in 19S and 20S proteasome subunits, 26S proteasome proteolytic activity is impaired leading to reduced protein turnover and increased cellular aggregate load.

### Proteasome dysfunction results in increased cell stress and death

**Figure 5:**
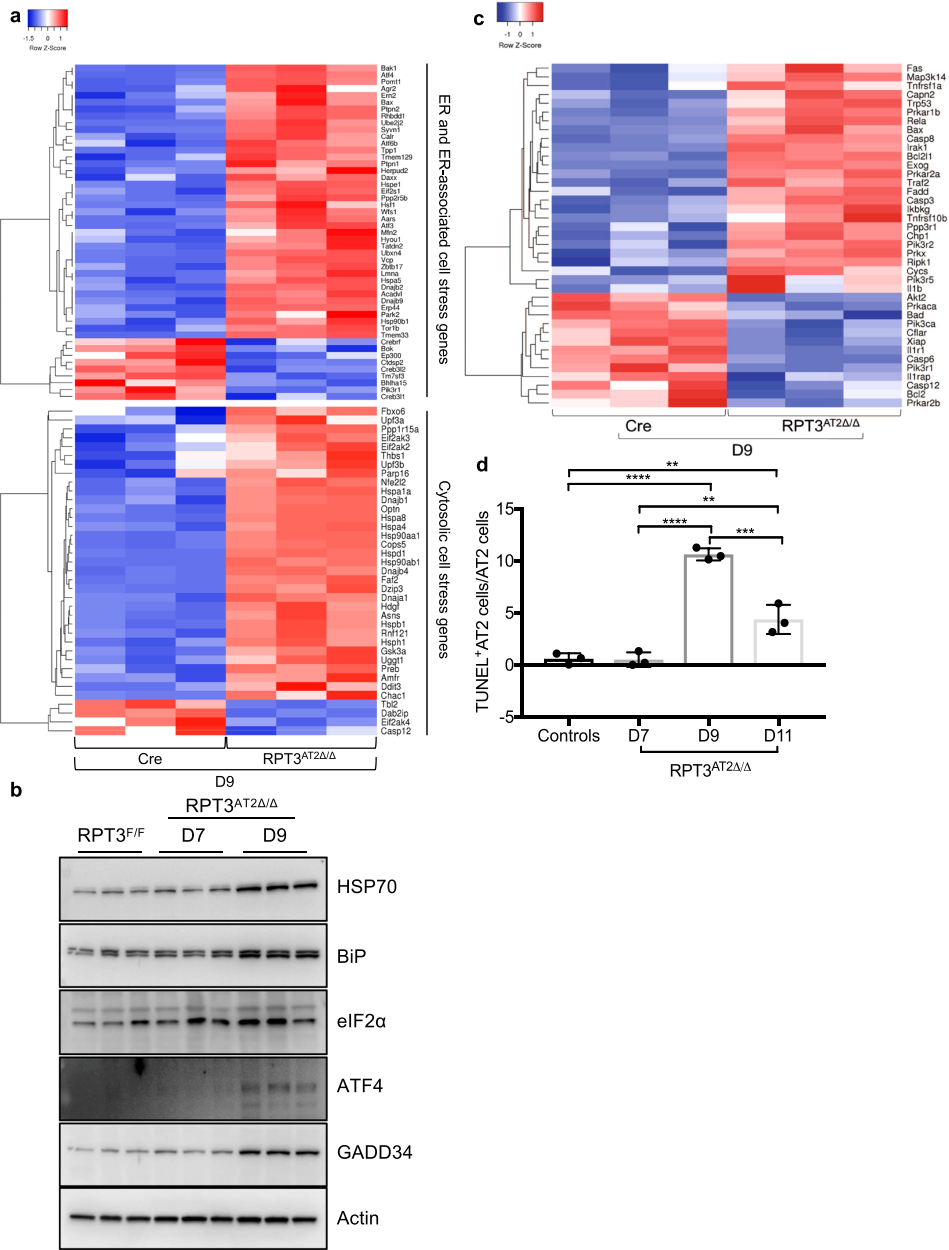
RPT3 deficiency results in increased cell stress and death. (**a**) Heatmap of differentially expressed cell stress response genes in AT2 cells isolated on D9. (**b**) Western blot analysis of cell stress markers in 30 μg of AT2 cell lysates separated by SDS-PAGE. (**c**) Heatmap of differentially expressed apoptosis-associated genes in AT2 cells isolated on D9. (**d**) Percentage of TUNEL^+^ AT2 cells assessed by morphometric analysis of Fig. S6a. Control included both RPT3^F/F^ mice (*Sftpc*^WT/CreER^:RPT3^F/F^ mice without tamoxifen treatment) and Cre mice that were fed tamoxifen chow for 7 days. Heatmaps were z-score normalized. Adjusted p-value < 0.05. n = 3 mice/genotype. *p < 0.05, **p < 0.01, ***p < 0.001, ****p < 0.0001 by one-way ANOVA with Tukey’s multiple comparison test. Full-length blots are presented in Supplementary Fig. 9.

Proteasome dysfunction triggers stress responses that allow cells to adapt to aggregate load or cause apoptosis^24^. AT2 cell-specific deletion of RPT3 resulted in global cell-stress response and was associated with upregulation of genes that encode heat shock proteins, ER chaperones and co-chaperones, and integrated stress response proteins (Fig. 5a); a comprehensive list of genes is provided in supplementary file 2. Western blot analyses of AT2 cells isolated on day 9 from RPT3^AT2Δ/Δ^ mice revealed an increase in cytosolic cell stress chaperone HSP70, ER stress chaperone BiP and downstream effectors of the integrated stress response, eIF2α, ATF4 and GADD34, compared to *Sftpc*^WT/CreER^:RPT3^F/F^ mice (Fig. 5b).

Significant enrichment of pro-apoptotic genes was observed after deletion of RPT3 (n = 26/39) (Fig. 5c). To confirm cell death, lung sections were stained with AT2 cell markers proSP-C and ABCA3, followed by terminal deoxynucleotidyl transferase dUTP nick end labelling (TUNEL) to detect apoptotic cells. Although TUNEL is not a conclusive indicator of apoptosis, necrosis was excluded based on histological analyses. The frequency of TUNEL^+^ AT2 cells was unchanged between controls (*Sftpc*^WT/CreER^:RPT3^F/F^ mice without tamoxifen and *Sftpc*^WT/CreER^ mice with tamoxifen treatment) and RPT3^AT2Δ/Δ^ mice (controls = 0.63 ± 0.5, RPT3^AT2Δ/Δ^ = 0.54 ± 0.7) on day 7 (Fig. 5d and Supplementary Fig. S6a). The frequency of TUNEL^+^ AT2 cells was increased by 17-fold (10.7 ± 0.6) and 7-fold (4.4 ± 1.4) on days 9 and 11, respectively, compared to control mice (Fig. 5d). The reduction in TUNEL^+^ AT2 cells on day 11 as compared to day 9 was consistent with the decreased number of AT2 cells on day 11 (Fig. 2c), indicating clearance of damaged AT2 cells. The frequency of total TUNEL^+^ nuclei (controls = 0.8 ± 0.3, D7 = 0.63 ± 1.0, D9 = 11.58 ± 2.7, D11 = 3.5 ± 2.4) was similar to that of TUNEL^+^ AT2 cells indicating that apoptosis was restricted to AT2 cells (Supplementary Fig. S6b). Collectively, these data indicate that accumulation of polyubiquitinated substrates is associated with unresolved cell stress and cell death.

### Autophagic activity is saturated in response to RPT3 deletion

**Figure 6:**
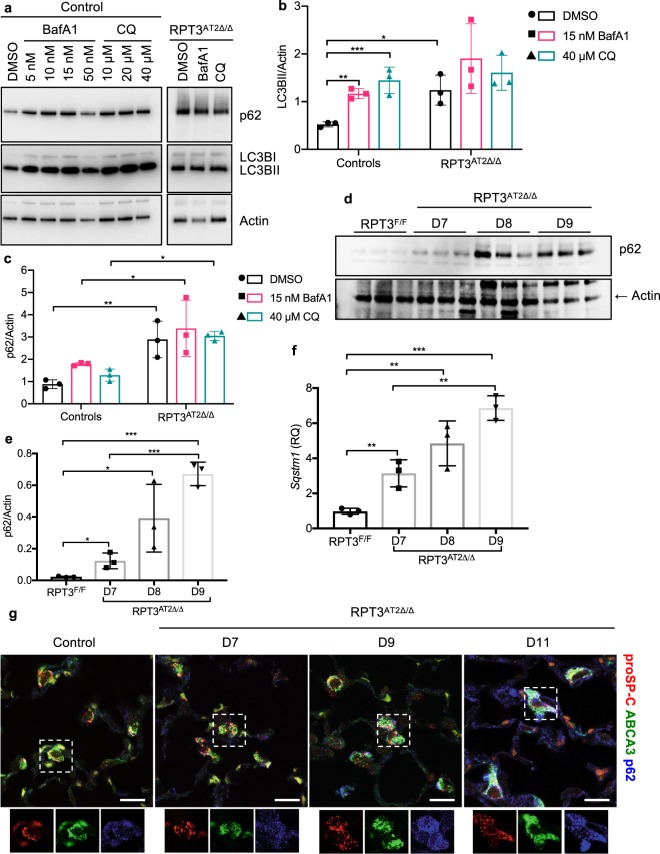
RPT3 deficiency induces p62 expression independent of autophagic activity. (**a**) Representative Western blot and densitometric analysis of LC3BII (**b**) and p62 (**c**) levels from three independent flux experiments. Samples and immunoblots were processed in parallel. Control included *Sftpc*^WT/CreER^:RPT3^F/F^ (RPT3^F/F^) mice without tamoxifen treatment or *Sftpc*^WT/CreER^ mice that were fed tamoxifen chow for 7 days. *p < 0.05, **p < 0.01, ***p < 0.001 by two-way ANOVA with Sidak’s multiple comparison test. (**d**) Western blot analysis and densitometry (**e**) of p62 in 30 μg of AT2 cell lysates separated by SDS-PAGE. (**f**) Quantitative PCR for *Sqstm1* (p62) in isolated AT2 cells. RQ: relative quantitation. *p < 0.05, **p < 0.01, ***p < 0.001 by one-way ANOVA with Tukey’s multiple comparison test. (**g**) Representative single focal plane image of lung sections stained for AT2 cells with proSP-C and ABCA3, and p62. Insets show individual channels for proSP-C, ABCA3, and p62. Scale bars = 10 μm. Confocal z-stacks were deconvoluted to obtain final images (details in methods). Controls included both RPT3^F/F^ mice (*Sftpc*^WT/CreER^:RPT3^F/F^ mice without tamoxifen treatment) and Cre mice that were fed tamoxifen chow for 7 days. Full-length blots are presented in Supplementary Fig. 9.

Increasing evidence suggests that autophagy can function as a backup system to help relieve proteasome overload^25–27^. To determine if basal autophagy was altered in response to proteasome dysfunction, LC3B levels were assessed by Western blotting. Despite upregulation of genes associated with the autophagy-lysosome pathway, (n = 113/176) (Supplementary Fig. S7a), LC3BI and lipidated LC3BII levels were not significantly altered in AT2 cells isolated from RPT3^AT2Δ/Δ^ mice compared to *Sftpc*^WT/CreER^:RPT3^F/F^ mice (Supplementary Fig. S7b–d). LC3B was primarily detected as the lipidated LC3BII form, consistent with previous data from isolated AT2 cells^28^. Further, expression of *Becn1*, an upstream regulator of autophagy^29^, was unaltered (Supplementary Fig. S7e), suggesting that deletion of RPT3 did not promote augmented autophagosome biogenesis at steady state. The latter hypothesis was tested by measuring autophagic flux *in vitro* using AT2 cells isolated from control mice (*Sftpc*^WT/CreER^:RPT3^F/F^ or tamoxifen treated *Sftpc*^WT/CreER^ mice) and RPT3^AT2Δ/Δ^ mice. In response to either Bafilomycin A1 or chloroquine, a significant increase was observed in LC3BII levels in control AT2 cells (DMSO: 0.524 ± 0.05, 15 nM Bafilomycin A1: 1.2 ± 0.11, 40 μM chloroquine: 1.5 ± 0.3) consistent with active autophagic flux (Fig. 6a,b). In contrast, LC3BII levels were already elevated in DMSO-treated AT2 cells isolated from RPT3^AT2Δ/Δ^ mice (1.2 ± 0.32), with no further increase in response to Bafilomycin A1 or chloroquine (15 nM Bafilomycin A1: 1.9 ± 0.73, 40 μM chloroquine: 1.6 ± 0.4) (Fig. 6a,b). Collectively, these data suggest that in response to deletion of RPT3 flux through the autophagy-lysosome system is near maximum, with little to no change in clearance of polyubiquitinated substrates.

### p62 fails to deliver substrates to the lysosome

Adaptor protein p62 targets aggregates to the autophagy-lysosome system and expression of p62 is rapidly upregulated in response to impaired proteasome activity^26,30,31^. Consistent with previous reports, p62 levels were increased by 5.2-fold in AT2 cells isolated from RPT3^AT2Δ/Δ^ mice on day 7 compared to *Sftpc*^WT/CreER^:RPT3^F/F^ mice (*Sftpc*^WT/CreER^:RPT3^F/F^ = 0.023 ± 0.004, RPT3^AT2Δ/Δ^ = 0.12 ± 0.05), by 17.4-fold (0.4 ± 0.2) on day 8 and by 30.4-fold (0.7 ± 0.07) on day 9 (Fig. 6d,e); additionally, accumulation of p62 protein was observed in surviving AT2 cells on day 11 (Fig. 6g). *Sqstm1* (p62) expression was upregulated by 3.2-fold (*Sftpc*^WT/CreER^:RPT3^F/F^ = 0.98 ± 0.18, RPT3^AT2Δ/Δ^ = 3.1 ± 0.8), 5-fold (4.9 ± 1.28) and 7-fold (6.9 ± 0.7) on days 7, 8 and 9, respectively, in AT2 cells isolated from RPT3^AT2Δ/Δ^ mice (Fig. 6f), consistent with RNA sequencing data (Supplementary File 2). Notably, p62 levels were significantly increased in AT2 cells isolated from RPT3^AT2Δ/Δ^ mice, irrespective of inhibitor treatment in the autophagic flux assay, compared to AT2 cells from control mice (Fig. 6a,c). Extensive accumulation of polyubiquitinated substrates and protein load was observed despite a significant increase in p62 and active autophagic flux. Since aggregates were detected by EM analysis in AT2 cells of RPT3^AT2Δ/Δ^ mice (Fig. 3o), we hypothesized that p62 was sequestered within ubiquitinated protein aggregates inhibiting transport for lysosomal degradation. To test this hypothesis, p62 was immunoprecipitated from AT2 cells isolated from *Sftpc*^WT/CreER^:RPT3^F/F^ and RPT3^AT2Δ/Δ^ mice on day 9 followed by Western blotting for total Ubiquitin. p62 preferentially associated with Ubiquitin in AT2 cells isolated from RPT3^AT2Δ/Δ^ mice compared to *Sftpc*^WT/CreER^:RPT3^F/F^ mice (Supplementary Fig. S8a). Additionally, p62 and K-48 conjugated ubiquitinated substrates were particularly enriched in detergent insoluble fraction obtained from lung homogenates of RPT3^AT2Δ/Δ^ mice compared to *Sftpc*^WT/CreER^:RPT3^F/F^ mice (Supplementary Fig. S8b). Collectively, these data suggest that proteasome dysfunction promotes an adaptive increase of p62 but sequestration of p62 within ubiquitinated aggregates inhibits substrate delivery to the lysosome for degradation.

## Discussion

RPT3 promotes assembly of the 19S particle with the 20S catalytic core to form active 26S proteasome. Partial deletion of RPT3 in AT2 cells resulted in dramatic accumulation of polyubiquitinated substrates that overwhelmed cellular disposal pathways leading to elevated cell stress and, ultimately, AT2 cell death. Rapid and extensive loss of AT2 cells was accompanied by depletion of alveolar surfactant and disruption of the alveolar epithelial barrier; the inability to rapidly re-epithelialize damaged alveoli resulted in lethal ARDS.

Formation of the 26S proteasome is dependent upon interactions between the C-terminal domain of 19S RPT subunits with 20S α subunits^32^. RPT subunits bind and hydrolyze ATP, which is required for substrate unfolding and 26S proteasome assembly and activation^13^; deletion of exons 7–11 of the *Psmc4* gene (RPT3) disrupts the AAA^+^-ATPase domain^20^. 26S proteasomes harboring ATP binding mutations in single RPT subunits have little effect on ATP hydrolyzing ability of the other five normal RPT subunits and can degrade small peptides, such as Suc-LLVY-AMC; however, these mutant proteasomes are much less efficient in degrading polyubiquitinated proteins^33^. RPT3 deletion resulted in accumulation of polyubiquitinated substrates, suggesting an inability to stimulate proteolytic activity of the 26S proteasome. Despite an adaptive increase in free 20S and 19S complexes, no apparent change was observed in 26S content or activity suggesting that loss of RPT3 resulted in *de novo* formation of few or unstable 26S proteasomes. Additionally, it is possible that aggregated proteins inhibited extant 26S proteasome function leading to further accumulation of potentially cytotoxic substrates.

p62 sequesters polyubiquitinated substrates into inclusions to protect the cell from cytotoxic aggregates; furthermore, p62 can deliver polyubiquitinated proteins to the autophagic machinery for clearance via the lysosome^34,35^. RPT3 deletion resulted in significant upregulation of p62 protein and mRNA, prior to accumulation of polyubiquitinated substrates. However, defective clearance of polyubiquitinated proteins despite increased lysosomal degradation suggests that sequestration of p62 within aggregates may impair substrate delivery to the lysosome resulting in augmented accumulation.

AT2 cell-specific deletion of RPT3 resulted in coincident increases in ubiquitinated substrate load, cell stress and apoptosis, consistent with a recent study published by Kitajima *et al*.^36^. These results are also consistent with the finding that treatment of cultured mouse AT2 cells with proteasome inhibitors resulted in dose-dependent cytotoxicity^7^. It is not clear if accumulation of ubiquitinated proteins is directly cytotoxic and/or if sequestration of regulatory proteins into aggregates leads to collapse of key cellular processes^37^. It is also possible that failure to degrade ubiquitinated substrates leads to depletion of essential amino acids resulting in induction of the integrated stress response^38^. Overall, the precise sequence of events between substrate accumulation and AT2 cell death remain unclear.

AT2 cells are facultative progenitors in the adult lung and respond to injury or loss by self-renewal and differentiation to AT1 cells^39^. RPT3^AT2Δ/Δ^ mice demonstrated respiratory failure following rapid loss of 90% AT2 cells over a 4-day period, with no evident compensatory proliferation. Deletion of RPT3 from muscle satellite cells inhibited proliferation in a p53-dependent manner and increased apoptosis^36^. Consistent with this report, a significant increase in *Trp53* and its associated pathways was observed in response to deletion of RPT3 from AT2 cells (supplementary file 2). Notably during the cell cycle, 26S proteasome is post-translationally modified, predominantly by phosphorylation of 19S and 20S subunits; inhibition of RPT3 phosphorylation resulted in 26S proteasome dysfunction, dysregulated degradation of cell cycle regulators and delayed cell cycle progression^40^. Collectively, these results support a model in which RPT3 deficiency inhibits proliferation of all or a subset of AT2 progenitors^41^, resulting in continual cell loss. The lethal ARDS-like phenotype observed in response to proteasome dysfunction in RPT3^AT2Δ/Δ^ mice is consistent with previous reports from AT2 cell-ablation models. Depletion of 80% AT2 cells over a 10-day period resulted in respiratory failure in SPC-TK mice^42^. Similarly, administration of diphtheria toxin to LysM-DTR mice resulted in lethality associated with depletion of AT2 cells^43^.

AT2-cell specific deletion of RPT3 resulted in acute morbidity and mortality associated with a 50% decrease in *Psmc4* (RPT3) expression. The lethal ARDS-like phenotype was associated with injury of both AT2 and AT1 cells, the latter likely secondary to loss of junctional complexes that anchor AT1 cells to AT2 cells; indeed, differential gene expression and GO analyses revealed downregulation of genes encoding tight junction proteins (supplementary file 2). Injury to the alveolar epithelial barrier was extensive compared to the underlying capillary endothelium. Alveolar surfactant pool size in RPT3^AT2Δ/Δ^ mice was decreased by 90% immediately prior to presentation of respiratory symptoms. A similar decrease in surfactant pool size was reported in response to deletion of ABCA3 in AT2 cells^44^; however, respiratory failure was delayed by more than a week in ABCA3 knockout mice. These findings provide further support for the hypothesis that extensive AT2 cell death and loss of barrier integrity are the primary drivers of acute respiratory failure in RPT3^AT2Δ/Δ^ mice; it is also likely that elevated surface tension arising from surfactant deficiency exacerbated AT2 cell injury and subsequent barrier dysfunction.

To date few studies have reported an association between proteasome dysfunction and ARDS: ubiquitin positive inclusion bodies were detected in alveolar epithelial cells of patients with diffuse alveolar damage^45^, and proteolytically active 20S proteasomes were released from damaged AT2 cells and recovered from BALF of patients with severe ARDS^46,47^. A recent case report identified complete alveolar denudation in two patients with diffuse alveolar damage and severe ARDS consistent with failure of AT2 cell-mediated lung repair^48^. In the current study, AT2 cell-specific deletion of *Psmc4* (RPT3) by 50% was sufficient to impair 26S proteasome function resulting in rapid AT2 cell apoptosis and lethal ARDS associated with an absence of AT2 cell proliferation and lung repair. Although the severe phenotype precludes the use of RPT3^AT2Δ/Δ^ mice as a clinical model of ARDS, this study underscores the importance of proteasome function in maintenance of AT2 cell homeostasis and supports the need to further investigate the role of proteasome dysfunction in ARDS pathogenesis.

## Methods

### Mice

RPT3^F/F^ mice on C57/Bl6 background were obtained from The Center for Animal Resources and Development (CARD), Kumamoto University, Japan^20^ and crossed with the *Sftpc*^CreERT2^ mice [on a mixed C57:129 genetic background^49^]. Genotyping was performed from tail biopsies; RPT3^F/F^ mice were genotyped with allele specific primers as described previously by Tashiro *et al*.^20^ and primers recommended by the Jackson Laboratory were used to distinguish between heterozygosity and homozygosity for the Cre allele. 8–12-week old female and male *Sftpc*^WT/CreER^:RPT3^F/F^ and *Sftpc*^WT/CreER^ control mice were provided *ad libitum* access to tamoxifen chow (400 mg tamoxifen citrate, Envigo). Mice were housed in a pathogen-free barrier facility. All procedures were performed under protocols (IACUC2015-0073: TEW) approved by the Institutional Animal Care and Use Committee of Cincinnati Children’s Hospital Medical Center in accordance with National Institutes of Health guidelines. Mice were euthanized when they demonstrated any of the following symptoms: kyphosis, severe lethargy and inactivity as observed by a lack of response when gently prodded, respiratory distress at rest as indicated by deep abdominal excursions, or weight loss of ≥20%.

### Alveolar type 2 (AT2) cell isolation

AT2 cells were isolated as previously described^44^. Briefly, lungs were perfused with 0.9% saline, followed by inflation with 2.5 ml dispase (BD Biosciences 354235). Single cell suspensions were prepared in C-tubes (Miltenyi Biotec 130-096-334) using the gentleMACS dissociator (Miltenyi Biotec) with 120 U/ml DNaseI (Sigma D4527). Suspensions were filtered through 40 μm strainers, resuspended in MACS buffer (1X PBS + 2 mM EDTA + 0.05% BSA) and incubated with the following anti-mouse biotinylated antibodies: CD45 (Biolegend 103104, clone 30-F11), CD16/32 (BD Pharmingen 553143, clone 2.462), CD31 (Biolegend 102503, clone MEC13.3), CD90.2 (Biolegend 105304, clone 30-H12) and Ter119 (Biolegend 116203, clone Ter119). Suspensions were subsequently incubated with anti-biotin microbeads (Miltenyi Biotec 130-097-046) and purified over a LS column (Miltenyi Biotec 130-042-401) attached to a QuadroMACS separator (Miltenyi Biotec). Eluates from the column (CD45^−^ CD16/32^−^ CD31^−^ CD90^−^ Ter119^−^) were stored at −80 °C as a dry pellet for Western blot analysis or processed immediately for RNA isolation.

### RNA isolation and quantitative real time (RT) PCR

Total RNA was isolated from 1 × 10^6^ AT2 cells using the Quick-RNA miniprep kit (Zymo Research R1054), and cDNA was synthesized using iScript cDNA Synthesis Kit (BioRad). Quantitative RT-PCR was performed with 25 ng of cDNA per reaction on the StepOne Plus Real Time PCR System (Thermo Fisher Scientific) or QuantStudio 3 (Thermo Fisher Scientific) with Taqman assays (Integrated DNA Technologies) for mouse *Psmc4* (RPT3; exons 3–4, Mm.PT.58.7882488 and exons 9–11, Mm.PT.58.12375476), *Psma5* (Mm.PT.58.30006634), *Psmb5* (Mm.PT.58.6715890), *Psmc3* (Mm.PT.58.43458789), *Psmd14* (Mm.PT.58.29611235), *Sqstm1* (Mm.PT.58.5854953) and *Becn1* (Mm.PT.58.31747082). Data were normalized to mouse Actin (*Actb*, Applied Biosystems, 4352933E).

### Western blot analysis

AT2 cells were harvested and lysed by sonication in 1X PBS containing 1% mammalian protease inhibitor cocktail (Sigma Aldrich) and 1X PhosSTOP (Sigma Aldrich) at a concentration of 1 × 10^6^ cells/80 μl. Protein concentration in the supernatants was assessed with the Pierce Micro BCA Kit (Thermo Fisher Scientific 23235). Equal amounts of proteins were separated on 10–20% tris-tricine gels (EC66252BOX, Thermo Fisher Scientific), or 10–20% tris-glycine gels (XP10202BOX, Thermo Fisher Scientific) under reducing conditions at 125 V for 1.5 hours and transferred to 0.1 μm nitrocellulose membranes (GE Amersham 10600000) or 0.2 μm Immobilon-PSQ PVDF membranes (EMD Millipore ISEQ. 10100) at 180 mA for 1 hour using a semi-dry apparatus. Membranes were blocked in 5% non-fat dry milk and subsequently incubated with primary antibodies (Supplementary Table S3) overnight at 4 °C. Membranes were incubated with appropriate HRP conjugated secondary antibodies (Supplementary Table S4), developed using Immobilon forte Western HRP substrate (EMD Millipore WBLUF0100) and analyzed on ChemiDoc Touch Imaging System (BioRad). Membranes were stripped with Restore Western blot stripping buffer (Thermo Fisher Scientific). Densitometric analysis was performed using ImageLab software (BioRad).

### Flow cytometric analysis

Lungs were inflated with 2.5 ml dispase, resected and incubated in dispase for 6 minutes at 37 °C. Lung lobes were separated and placed in C-tubes containing 5 ml DMEM (Life Tech 11995–065) with 1 M HEPES and Penicillin/Streptomycin. Single cell suspensions were prepared using the gentleMACS dissociator with 120 U/ml DNaseI and filtered through a 40 μm strainer, washed with media and incubated in RBC lysis buffer (BioLegend 420301) for 4 minutes at 4 °C. Suspensions were washed with media, cell counts were performed and suspended at a concentration of 1 × 10^6^ cells/100 μl in FACS buffer (1X PBS + 5% FBS + 5 mM EDTA), and incubated with CD16/32 (BD Pharmingen 553142, clone 2.4G2) for 15 minutes at 4 °C. Cells were washed with FACS buffer and incubated with the following antibodies for 1 hour at 4 °C: AF700 CD45 (BioLegend 103128, clone 30-F11), PE CD31 (eBioscience 12–0311–81, clone 390) and APC/Cy7 CD326 (BioLegend 118217, clone G8.8). Cells were repeatedly washed with PBS and incubated with viability dye (BD Biosciences 566332 440UV fixable viability stain) for 15 minutes at 4 °C, followed by fixation with the FoxP3 transcription factor staining buffer kit (eBioscience 00–5523–00) for 20 minutes at room temperature. Cells were resuspended in FACS buffer for flow cytometric analysis. Data were acquired using the LSR Fortessa 1 flow cytometer (BD Biosciences) and analyzed with FlowJo software (FlowJo, LLC). Compensation was performed with OneComp ebeads (eBioscience 01-1111-42) according to manufacturer’s instructions.

### Histology, immunofluorescence and TUNEL assay

Lungs were inflated with 4% paraformaldehyde under 25 cm pressure and immersed in the same fixative overnight at 4 °C. Right lung lobes and left lung were sub-dissected and embedded in paraffin after dehydration in an ethanol series. Lung pieces were sectioned at 5 μm using a Leica RM2235 microtome for hematoxylin and eosin (H&E) staining. Tile scans of H&E stained sections were obtained using a Nikon NiE upright microscope. For immunofluorescence analyses, sections were stained with primary antibodies (Supplementary Table S3) with standard sodium citrate antigen retrieval as required. All Alexa-fluor conjugated secondary antibodies (Supplementary Table S4) were used at a dilution of 1:200. TUNEL assay (Roche 11684817910) was performed according to manufacturer’s instructions on immunofluorescent labelled lung sections. High magnification images were taken at 60X with Nyquist magnification using Nikon A1 LUNA inverted microscope or at 100X using Nikon A1R LUNV inverted microscope. Confocal images in Fig. 6 were deconvoluted using Lucy-Richardson algorithm with 15 iterations on Nikon Elements.

### Morphometric analysis

Twenty random 20X fields per mouse were imaged on a Nikon NiE upright microscope. General analysis program on Nikon Elements was used to perform morphometric analysis. Each marker was separated into an individual spectral channel (FITC, TRITC or Cy5) after correcting for autofluorescence. Bright spot detection was used to identify individual cells or nuclei of interest with constant diameter and a contrast range across image frames [DAPI, diameter = 7.5 μm, contrast = 167.5 (constant); TUNEL-FITC, diameter = 5.6 μm, contrast = 98.20–288.6; proSP-C, diameter = 9.32 μm, contrast (TRITC) = 18.04–41.9, contrast (Cy5) = 41.88–137.6; ABCA3, diameter = 9.32 μm, contrast (TRITC) = 41.88–106.4 contrast (Cy5) = 29.82–62.21]. Each marker spot was counted based on its overlap with a DAPI^+^ nucleus. Double and triple positive cells were counted based on overlap of each individual marker and a DAPI^+^ nucleus. The number of AT2 cells per frame was defined as the sum of proSP-C^+^ ABCA3^−^, proSP-C^−^ ABCA3^+^ and proSP-C^+^ ABCA3^+^ cells divided by the number of DAPI^+^ nuclei per image frame. The average of twenty fields is shown for each individual mouse. A total of 25,000–30,000 DAPI^+^ nuclei were counted per mouse. For control mice, 3000–5000 AT2 cells were counted per mouse.

### Bronchoalveolar lavage fluid (BALF) collection, cell differentials and surfactant lipid measurements

BALF was collected by intratracheal intubation and 5 serial lung lavages using 1 ml 0.9% saline. Immune cells were isolated from BALF by centrifugation at 230 g for 10 minutes, and pellets were resuspended in 100 μl of 0.9% saline for total and differential cell analyses. Cell-free BALF was used for measurement of surfactant lipids and total protein. Lipids were extracted from cell-free BALF by the Bligh and Dyer method^50^. Saturated phosphatidylcholine was isolated using the osmium tetroxide based method of Mason *et al*.^51^ and quantitated by phosphorous measurement. Cytospin slides were prepared at a cell density of 1 × 10^6^ cells/ml and stained with the Shandon Kwik-Diff stain kit (Thermo Fisher Scientific). A total of 300 cells was counted manually per slide to determine the percentage of monocyte/macrophages, lymphocytes and neutrophils.

### Transmission electron microscopy

Mouse lungs were inflation-fixed with 2% paraformaldehyde [Electron Microscopy Sciences (EMS)], 2% glutaraldehyde (EMS), 0.1% calcium chloride (Fisher Scientific) in 0.1 M sodium cacodylate (EMS) buffer (SCB), pH 7.2, under 25 cm pressure, followed by immersion fixation with fresh fixative at 4 °C overnight. Lung lobes were cut into 1–2 mm blocks and processed for transmission electron microscopy as previously described^52^. 90 nm mouse lung sections were viewed, and images were digitally acquired by a Hitachi H-7650 transmission electron microscope (Hitachi High Technologies USA) equipped with a CCD camera (Advanced Microscopy Techniques) at 80 kV.

### Scanning electron microscopy

Paraffin embedded mouse lung blocks used for confocal microscopy were dewaxed, rehydrated, and processed for scanning electron microscopy. Dewaxed mouse lung blocks were washed with HemoDe^®^ (EMS) to remove excess paraffin, followed by rehydration through a graded series of alcohol and distilled water mixtures^53^. To enhance specimen contrast and surface conductivity, rehydrated mouse lung blocks were first incubated with 1% osmium tetraoxide (OsO_4_; EMS) and 1.5% potassium ferrocyanide (EMS) in 0.1 M SCB, pH 7.2, at room temperature for 2 hours, rinsed with 0.1 M SCB, incubated with freshly prepared 1% thiocarbohydrazide (Sigma) for 30 minutes, and fresh 1% OsO_4_ in 0.1 M SCB for an additional hour. After osmium impregnation, *en bloc* staining with 4% uranyl acetate (EMS) was conducted at 4 °C overnight, followed by freshly prepared Walton’s lead aspartate staining *en bloc* at 60 °C for 1 hour^54,55^. Lung blocks were dehydrated through a series of graded alcohol and processed for critical drying using a Leica EM critical point dryer (Leica Microsystems Inc.). Mouse lung samples were mounted on 15 mm specimen stubs (EMS) and viewed with a Hitachi SU-8010 field emission scanning electron microscope (Hitachi High Technologies USA) at 5 kV without further coating.

### Barrier permeability measurements

Epithelial barrier permeability was measured as previously described^56^. Briefly, mice were intravenously injected with 300 μl of 12 mg/ml albumin conjugated to FITC (Sigma-Aldrich A9771), under 2% isoflurane anesthesia. BALF was recovered after 2 hours by lavage with 1 ml of 0.9% saline, and whole blood was collected via cardiac puncture. FITC fluorescence in BALF and serum was determined using the Synergy2 Multimode Microplate Reader (BioTek) with absorption/emission wavelengths of 480/520 nm. Barrier permeability was defined as the ratio of fluorescence in BALF to fluorescence in serum.

### RNA sequencing and analysis

Total RNA was extracted from the entire pool of freshly isolated AT2 cells using RNeasy mini prep kit (Qiagen) and sent to Novogene Corporation (Chula Vista, CA) for RNA sequencing. mRNA was purified from total RNA (RNA integrity number >9) and sequencing libraries were generated using NEBNext^®^ Ultra^™^ RNA library prep kit for Illumina. Sequencing was carried out on Illumina HiSeq 4000 platform and paired-end reads were generated. Filtered reads were aligned to the mouse reference genome mm10 using STAR (version 2.5). Differential expressions were determined through DESeq2 R package (version 2_1.6.3). Resulting p-values were adjusted using Benjamini and Hochberg’s method for controlling false discovery rate. Genes with an adjusted p-value < 0.05 were assigned as differentially expressed. Gene ontology (GO) analysis was performed using Toppfun on Toppgene suite (https://toppgene.cchmc.org/) with Bonferroni method for multiple correction and 0.05 cut-off for significance, or clusterProfiler R package (version 2.4.3) with 0.05 cut-off for adjusted p-value. Heatmaps were generated using Heatmapper (http://www2.heatmapper.ca/expression/).

### Native gel analysis and proteasome activity assay

Native gel electrophoresis and proteasome activity assay were performed as previously described^14,57^. Briefly, isolated AT2 cells were lysed with ice cold lysis buffer (50 mM Tris-HCl, pH 7.5, 0.5% NP-40, 1 mM DTT, 2 mM ATP, and 5 mM MgCl_2_) at a concentration of approximately 1 × 10^6^ cells/100 μl by repeated pipetting. Protein concentration in the cell lysates was determined using the micro BCA kit. Equal amounts of protein were separated on 3–8% tris acetate gels (EA0378BOX, Life Technologies) at 150 V for 4 hours at 4 °C. Gels were incubated for 30 minutes at 37 °C in assay buffer (50 mM Tris, pH 7.5, 10 mM MgCl_2_, 1 mM ATP, 1 mM DTT) containing 50 μM Suc-LLVY-AMC (Sigma S6510) to monitor chymotrypsin-like activity of the 26S proteasome. Gels were imaged on Chemidoc touch imaging system with a UV transilluminator (AMC excitation/emission wavelengths are 380/460 nm). Subsequently, SDS was added to a final concentration of 0.02% and gels were incubated in assay buffer for an additional 20 minutes at 37 °C to image the latent activity of the 20S catalytic core. Gels were washed briefly in 50 mM Tris, pH 7.5 and incubated in 2% SDS, 66 mM Na_2_CO_3_, 1.5% β-mercaptoethanol for 10 minutes prior to semi-dry transfer to Immobilon-PSQ PVDF membranes at 250 mA for 1.5 hours. Proteasome complexes were immunoblotted as described in the Western blotting section. Imperial protein stain (Thermo Fisher Scientific 24615) was performed to assess protein loading. Human 26S proteasome (Enzo Life Sciences BML-PW9310-0050) and bovine 20S proteasome (UBPBio A1400) were used as controls.

### Autophagy flux assay

AT2 cells were isolated as described above and cultured with BEGM media (containing all supplements except hydrocortisone, Lonza CC-3170) with 5% charcoal stripped FBS (Life Technologies 12676-011) on 100% Cultrex (R&D 3433-005-01) coated plates for 48 hours. 1 × 10^6^ AT2 cells were incubated with Bafilomycin A1 (Invivogen tlrl-baf1) or chloroquine (Invivogen tlrl-chq). Cultrex was dissolved with Cell Recovery Solution (Corning 354253) according to manufacturer’s instructions and cells were collected in 2X sample buffer containing BME. Samples were separated on 10–20% tris-tricine gels and analyzed by Western blotting.

### p62 immunoprecipitation and fractionation

p62 was immunoprecipitated from freshly isolated AT2 cells as previously described^26^. Briefly, cells were lysed in 150 mM NaCl, 10 mM Tris-HCl (pH 7.5), 1% NP-40, supplemented with 1% protease inhibitors. 200 μg of protein was pre-cleared with Sepharose A beads (Invitrogen 101141) for 3 hours. Pre-cleared lysate was incubated with rabbit p62 antibody (Sigma-Aldrich P0067) or rabbit serum (Sigma R9133) for 16 hours. Antibody bound lysates were subsequently incubated with Sepahrose A beads for 30 minutes. Beads were washed 4 times with 150 mM NaCl, 10 mM Tris-HCl (pH 7.5), 1% NP-40, and resuspended in 2X sample buffer containing BME for SDS-PAGE/Western blot analysis. For analysis of detergent insoluble p62 the right lower lung lobe was homogenized in 150 mM NaCl, 10 mM Tris-HCl (pH 7.5) and 1% NP-40, supplemented with 1% protease inhibitors using the Qiagen TissueLyser II system. Insoluble material was pelleted by centrifugation at 16,000 g for 15 min at 4 °C and resuspended by sonication in 150 mM NaCl, 10 mM Tris-HCl (pH 7.5), 2% SDS. Proteins were quantified in both pre- and post-spin lysates and 30 μg were resolved on 10–20% tris-glycine gel for Western blotting analyses.

### Statistics

Statistical analysis was performed using GraphPad Prism (GraphPad Software). All data are presented as mean ± SD. Statistical tests used for comparison of data are reported in the respective figure legends.

## Supplementary information

Supplementary file 1

Dataset 1

## Data Availability

RNA sequencing data is available on GEO (GSE123873). List of all differentially expressed genes and GO terms used to generate heatmaps are provided in supplementary file 2.

## References

[1] Zhao J, Goldberg AL (2016). Coordinate regulation of autophagy and the ubiquitin proteasome system by MTOR. Autophagy.

[2] Tsakiri EN, Trougakos IP (2015). The amazing ubiquitin-proteasome system: structural components and implication in aging. Int Rev Cell Mol Biol.

[3] Weathington NM, Mallampalli RK (2014). Emerging therapies targeting the ubiquitin proteasome system in cancer. J Clin Invest.

[4] Weiss CH, Budinger GR, Mutlu GM, Jain M (2010). Proteasomal regulation of pulmonary fibrosis. Proc Am Thorac Soc.

[5] Mutlu GM (2012). Proteasomal inhibition after injury prevents fibrosis by modulating TGF-beta(1) signalling. Thorax.

[6] Fineschi S (2008). *In vivo* investigations on anti-fibrotic potential of proteasome inhibition in lung and skin fibrosis. Am J Respir Cell Mol Biol.

[7] Semren N (2015). Validation of the 2nd Generation Proteasome Inhibitor Oprozomib for Local Therapy of Pulmonary Fibrosis. PLoS One.

[8] Li J, Chen S, Hu Y, Cai J (2016). Bortezomib-induced severe pulmonary complications in multiple myeloma: A case report and literature review. Oncol Lett.

[9] Yoshizawa K (2014). Bortezomib therapy-related lung disease in Japanese patients with multiple myeloma: Incidence, mortality and clinical characterization. Cancer Science.

[10] Kang W (2010). Nonspecific Interstitial Pneumonitis after Bortezomib and Thalidomide Treatment in a Multiple Myeloma Patient. Yonsei Medical Journal.

[11] Dhakal A, Belur AA, Chandra AB (2014). Bortezomib Induced Pulmonary Toxicity. Blood.

[12] Balsman E (2016). Bortezomib therapy-related lung disease in a patient with light chain amyloidosis: A case report. Journal of Oncology Pharmacy Practice.

[13] Kim YC, Li X, Thompson D, DeMartino GN (2013). ATP binding by proteasomal ATPases regulates cellular assembly and substrate-induced functions of the 26 S proteasome. J Biol Chem.

[14] Kaneko T (2009). Assembly pathway of the Mammalian proteasome base subcomplex is mediated by multiple specific chaperones. Cell.

[15] Murata S, Yashiroda H, Tanaka K (2009). Molecular mechanisms of proteasome assembly. Nat Rev Mol Cell Biol.

[16] Kusmierczyk AR, Kunjappu MJ, Kim RY, Hochstrasser M (2011). A conserved 20S proteasome assembly factor requires a C-terminal HbYX motif for proteasomal precursor binding. Nat Struct Mol Biol.

[17] Gomes AV (2013). Genetics of proteasome diseases. Scientifica (Cairo).

[18] Verheijen, B. M., Oyanagi, K. & van Leeuwen, F. W. Dysfunction of Protein Quality Control in Parkinsonism–Dementia Complex of Guam. *Frontiers in Neurology* **9**, 10.3389/fneur.2018.00173 (2018). 10.3389/fneur.2018.00173 PMC5869191 29615966

[19] Grünblatt E (2018). Differential Alterations in Metabolism and Proteolysis-Related Proteins in Human Parkinson’s Disease Substantia Nigra. Neurotoxicity Research.

[20] Tashiro Y (2012). Motor neuron-specific disruption of proteasomes, but not autophagy, replicates amyotrophic lateral sclerosis. J Biol Chem.

[21] Kitajima Y (2014). Proteasome dysfunction induces muscle growth defects and protein aggregation. J Cell Sci.

[22] Sakao Y (2000). Mouse proteasomal ATPases Psmc3 and Psmc4: genomic organization and gene targeting. Genomics.

[23] Parra E, Perez-Gil J (2015). Composition, structure and mechanical properties define performance of pulmonary surfactant membranes and films. Chem Phys Lipids.

[24] Flick K, Kaiser P (2012). Protein degradation and the stress response. Seminars in cell & developmental biology.

[25] Houck SA (2014). Quality control autophagy degrades soluble ERAD-resistant conformers of the misfolded membrane protein GnRHR. Mol Cell.

[26] Milan E (2015). A plastic SQSTM1/p62-dependent autophagic reserve maintains proteostasis and determines proteasome inhibitor susceptibility in multiple myeloma cells. Autophagy.

[27] Kageyama S (2014). Proteasome dysfunction activates autophagy and the Keap1-Nrf2 pathway. J Biol Chem.

[28] Hahn DR, Na CL, Weaver TE (2014). Reserve autophagic capacity in alveolar epithelia provides a replicative niche for influenza A virus. Am J Respir Cell Mol Biol.

[29] Ryter SW, Choi AM (2015). Autophagy in lung disease pathogenesis and therapeutics. Redox Biol.

[30] Acosta-Alvear D (2015). Paradoxical resistance of multiple myeloma to proteasome inhibitors by decreased levels of 19S proteasomal subunits. Elife.

[31] Sha Z, Schnell HM, Ruoff K, Goldberg A (2018). Rapid induction of p62 and GABARAPL1 upon proteasome inhibition promotes survival before autophagy activation. J Cell Biol.

[32] Kumar B, Kim YC, DeMartino GN (2010). The C terminus of Rpt3, an ATPase subunit of PA700 (19S) regulatory complex, is essential for 26S proteasome assembly but not for activation. The Journal of biological chemistry.

[33] Liu CW (2006). ATP binding and ATP hydrolysis play distinct roles in the function of 26S proteasome. Mol Cell.

[34] Demishtein A (2017). SQSTM1/p62-mediated autophagy compensates for loss of proteasome polyubiquitin recruiting capacity. Autophagy.

[35] Zaffagnini, G. *et al*. p62 filaments capture and present ubiquitinated cargos for autophagy. *EMBO J* **37**, 10.15252/embj.201798308 (2018). 10.15252/embj.201798308 PMC5830917 29343546

[36] Kitajima Y (2018). The Ubiquitin-Proteasome System Is Indispensable for the Maintenance of Muscle Stem Cells. Stem Cell Reports.

[37] Yu A (2014). Protein aggregation can inhibit clathrin-mediated endocytosis by chaperone competition. Proc Natl Acad Sci USA.

[38] Suraweera A, Munch C, Hanssum A, Bertolotti A (2012). Failure of amino acid homeostasis causes cell death following proteasome inhibition. Mol Cell.

[39] Barkauskas CE (2013). Type 2 alveolar cells are stem cells in adult lung. J Clin Invest.

[40] Guo X (2016). Site-specific proteasome phosphorylation controls cell proliferation and tumorigenesis. Nat Cell Biol.

[41] Zacharias WJ (2018). Regeneration of the lung alveolus by an evolutionarily conserved epithelial progenitor. Nature.

[42] Garcia O (2016). Targeted Type 2 Alveolar Cell Depletion. A Dynamic Functional Model for Lung Injury Repair. Am J Respir Cell Mol Biol.

[43] Miyake Y (2007). Protective role of macrophages in noninflammatory lung injury caused by selective ablation of alveolar epithelial type II Cells. J Immunol.

[44] Rindler, T. N. *et al*. Alveolar injury and regeneration following deletion of ABCA3. *JCI Insight* **2**, 10.1172/jci.insight.97381 (2017). 10.1172/jci.insight.97381 PMC5752264 29263307

[45] Yamada T (2006). Immunohistochemical detection of ubiquitin-positive intracytoplasmic eosinophilic inclusion bodies in diffuse alveolar damage. Histopathology.

[46] Sixt SU (2009). Alveolar extracellular 20S proteasome in patients with acute respiratory distress syndrome. Am J Respir Crit Care Med.

[47] Sixt SU, Peters J (2010). Extracellular Alveolar Proteasome..

[48] Taylor MS (2018). A Conserved Distal Lung Regenerative Pathway in Acute Lung Injury. Am J Pathol.

[49] Rock JR (2011). Multiple stromal populations contribute to pulmonary fibrosis without evidence for epithelial to mesenchymal transition. Proceedings of the National Academy of Sciences of the United States of America.

[50] Bligh EG, Dyer WJ (1959). A rapid method of total lipid extraction and purification. Canadian journal of biochemistry and physiology.

[51] Mason RJ, Nellenbogen J, Clements JA (1976). Isolation of disaturated phosphatidylcholine with osmium tetroxide. Journal of lipid research.

[52] Yang L (2017). The Phosphatidylcholine Transfer Protein Stard7 is Required for Mitochondrial and Epithelial Cell Homeostasis. Sci Rep.

[53] Widéhn, S.G. & Kindblom, L. A rapid and simple method for electron microscopy of paraffin-embedded tissue. Vol. 12 (1988). 10.3109/01913128809048481 3354071

[54] Tapia JC (2012). High-contrast en bloc staining of neuronal tissue for field emission scanning electron microscopy. Nature protocols.

[55] Walton J (1979). Lead aspartate, an en bloc contrast stain particularly useful for ultrastructural enzymology. The journal of histochemistry and cytochemistry: official journal of the Histochemistry Society.

[56] Yang L (2015). Haploinsufficiency for Stard7 is associated with enhanced allergic responses in lung and skin. J Immunol.

[57] Semren N (2015). Regulation of 26S Proteasome Activity in Pulmonary Fibrosis. Am J Respir Crit Care Med.

