# Supplementary material for: Proteasome dysfunction in alveolar type 2 epithelial cells is associated with acute respiratory distress syndrome
Source: Sci Rep. 2019 Aug 29;9:12509. doi: 10.1038/s41598-019-49020-4 (PMC6715642; doi:10.1038/s41598-019-49020-4)
Supplement: Supplementary file 1 [file 41598_2019_49020_MOESM1_ESM.pdf]

Proteasome dysfunction in alveolar type 2 epithelial cells is associated with acute respiratory distress syndrome

Sneha Sitaraman, Cheng-Lun Na, Li Yang, Alyssa Filuta, James P. Bridges, Timothy E. Weaver

Figure S1: Tamoxifen treatment strategies for RPT3$^{F/F}$ mice.

a. Weight loss of RPT3$^{AT2\Delta/\Delta}$ and *Sftpc*$^{WT/CreER}$ (Cre) mice on tamoxifen chow. Mice were fed tamoxifen chow for 7 days and monitored daily. Starting weights of mice were normalized to 100%. **b.** Quantitative PCR for *Psmc4* (RPT3) exons 3-4 in AT2 cells isolated after 7 days of tamoxifen treatment. Primer probe set directed to exons 3-4 is upstream of the targeted region (exons 7-11). RQ: relative quantitation. **c.** Kaplan Meier survival curve for mice placed on tamoxifen chow for indicated number of days (indicated in figure label). **d.** Schematic of study for e-f. **e & f.** Quantitative PCR for *Psmc4* (RPT3) exons 9-11 and Western blot analysis of AT2 cells isolated from RPT3$^{F/F}$ mice that were fed tamoxifen chow for 4 days. Note that *Psmc4* expression and RPT3 levels were not statistically different compared to controls. ns: not significant. RPT3$^{F/F}$: *Sftpc*$^{WT/CreER}$:RPT3$^{F/F}$ mice without tamoxifen treatment.

Figure S2: AT2 cell-specific deletion of RPT3 results in loss of lung epithelial cells and mild inflammation.

a. Gating strategy for flow cytometric analysis and frequency of live CD326^{+} cells. **$p<0.01$ by t-test. **b-d.** Representative tile scans of H&E stained left lung lobes. Arrowhead points to perivascular inflammation in d'. Scale bars= 500 μm (b-d) and 50 μm (d'). n= 6-8 mice/timepoint. RPT3$^{F/F}$: *Sftpc*$^{WT/CreER}$:RPT3$^{F/F}$ mice without tamoxifen treatment.

Figure S3. AT1 cell damage in RPT3$^{AT2\Delta/\Delta}$ mice.

a. Representative maximum intensity projections of confocal z-stacks of lung sections stained with T1α (Podoplanin). Arrowheads point to distended AT1 cells and arrows point to T1α^{+} debris. **b.** Representative maximum intensity projections of confocal z-stacks of

lung sections stained with EMCN (Endomucin). Br: bronchiole, AD: alveolar duct, Alv: alveolus. Scale bar= 50 μm. n= 2-3 mice/genotype. Controls included both RPT3$^{F/F}$ mice (*Sftpc*$^{WT/CreER}$:RPT3$^{F/F}$ mice without tamoxifen treatment) and Cre mice that were fed tamoxifen chow for 7 days.

Figure S4. Altered epithelial barrier permeability in RPT3$^{AT2\Delta/\Delta}$ mice.

a. FITC-albumin was intravenously injected through the tail vein on day 11, and BALF was collected 2 hours after injection. FITC fluorescence measured in BALF was normalized to FITC fluorescence measured in serum. **b.** Protein concentration measured in cell-free BALF obtained on day 11. Independent groups of mice were used for experiments in a & b. **p<0.01 by one-way ANOVA with Tukey's multiple comparison test. **c.** Cell differential of immune cells recovered from BALF obtained from mice in b. Data is represented as a percentage of total cells counted. **d.** Total number of immune cells recovered from BALF obtained from mice in b. *p<0.05, **p<0.01 by two-way ANOVA with Sidak's multiple comparison test. RPT3$^{F/F}$: *Sftpc*$^{WT/CreER}$:RPT3$^{F/F}$ mice without tamoxifen treatment.

Figure S5. RPT3 deficiency results in upregulation of proteasome-associated genes.

a. Volcano plot of all differentially expressed genes in AT2 cells isolated RPT3$^{AT2\Delta/\Delta}$ mice from compared to *Sftpc*$^{WT/CreER}$ (Cre) mice. **b.** Heatmap of differentially regulated proteasome-associated genes and subunits **(c)** in AT2 cells isolated on D9. Heatmaps were z-score normalized. Adjusted p-value (padj) <0.05. n=3 mice/genotype. **d.** Quantitative PCR for proteasome subunits *Psma5*, *Psmb5*, *Psmc3* and *Psmd14* in isolated AT2 cells. Samples unrelated to RNA sequencing were used to validate data. **e.**

Quantitative PCR for *Psmc4* (RPT3) exons 3-4 and exons 9-11 in AT2 cells. Primer probe set directed to exons 3-4 is upstream of the targeted region (exons 7-11). Samples submitted for RNA sequencing and an independent set of samples were used for validation of RNA sequencing data. RQ: relative quantitation **f.** Western blot analysis and densitometry **(g)** of RPT3 in 30 μg of AT2 cell lysates separated by SDS-PAGE. *p<0.05, **p<0.01, ***p<0.001, ****p<0.0001 by one-way ANOVA with Tukey's multiple comparison test. RPT3$^{F/F}$: *Sftpc*$^{WT/CreER}$:RPT3$^{F/F}$ mice without tamoxifen treatment.

Figure S6. RPT3 deficiency results in cell death.

a. Representative maximum intensity projections of confocal z-stacks of lung sections stained for AT2 cells with proSP-C and ABCA3, and TUNEL. Insets show a proSP-C$^+$ ABCA3$^+$ cell and a TUNEL$^+$ AT2 nucleus (D9 and D11). Scale bars= 50 μm. **b.** Percentage of all TUNEL$^+$ nuclei determined by morphometric analysis of lung sections from a. Controls included both RPT3$^{F/F}$ mice (*Sftpc*$^{WT/CreER}$:RPT3$^{F/F}$ mice without tamoxifen treatment) and Cre mice that were fed tamoxifen chow for 7 days. *p<0.05, **p<0.01, ***p<0.001 by one-way ANOVA with Tukey's multiple comparison test.

Figure S7. Autophagosome synthesis is unaltered at steady state in response to RPT3 deficiency.

a. Heatmap of differentially expressed autophagy-associated genes in AT2 cells isolated on D9. Heatmaps were z-score normalized. Adjusted p-value <0.05 n=3 mice/genotype. **b-d.** Western blot analysis and densitometry of LC3B in 30 μg of AT2 cell lysates separated by SDS-PAGE. LE: long exposure. SE: short exposure. **e.** Quantitative PCR for *Becn1* in isolated AT2 cells. Samples unrelated to RNA sequencing were used to verify

Becn1 expression. RQ: relative quantitation. RPT3$^{F/F}$: *Sftpc*$^{WT/CreER}$:RPT3$^{F/F}$ mice without tamoxifen treatment.

Figure S8. p62 is sequestered within ubiquitinated aggregates.

a. p62 was immunoprecipitated from AT2 cell lysates followed by Western blotting for Ubiquitin. Immunoprecipitation was verified using a p62 antibody generated in mouse (Ms). Lysates were incubated with rabbit (Rb) serum as control for antibody binding (- lanes). Lysis buffer was incubated with p62 antibody to exclude non-specific protein association. IgG-H: immunoglobulin heavy chain. Samples are from the same experiment. Two different exposures are shown to distinguish signals and exclude bleed through. **b.** Western blot analysis of p62 and Ubiquitin in detergent soluble (pre-spin and post-spin) and insoluble (NP-40 ins.) fractions obtained from lung homogenates. AT2 cells for panel A and lungs for panel B were obtained from RPT3$^{AT2\Delta/\Delta}$ mice on day 9. RPT3$^{F/F}$: *Sftpc*$^{WT/CreER}$:RPT3$^{F/F}$ mice without tamoxifen treatment.

Figure S9. Uncropped immunoblots.

Figure numbers and proteins are provided with respective blots. Bands of interest are marked by boxes.

Supplementary figure 1

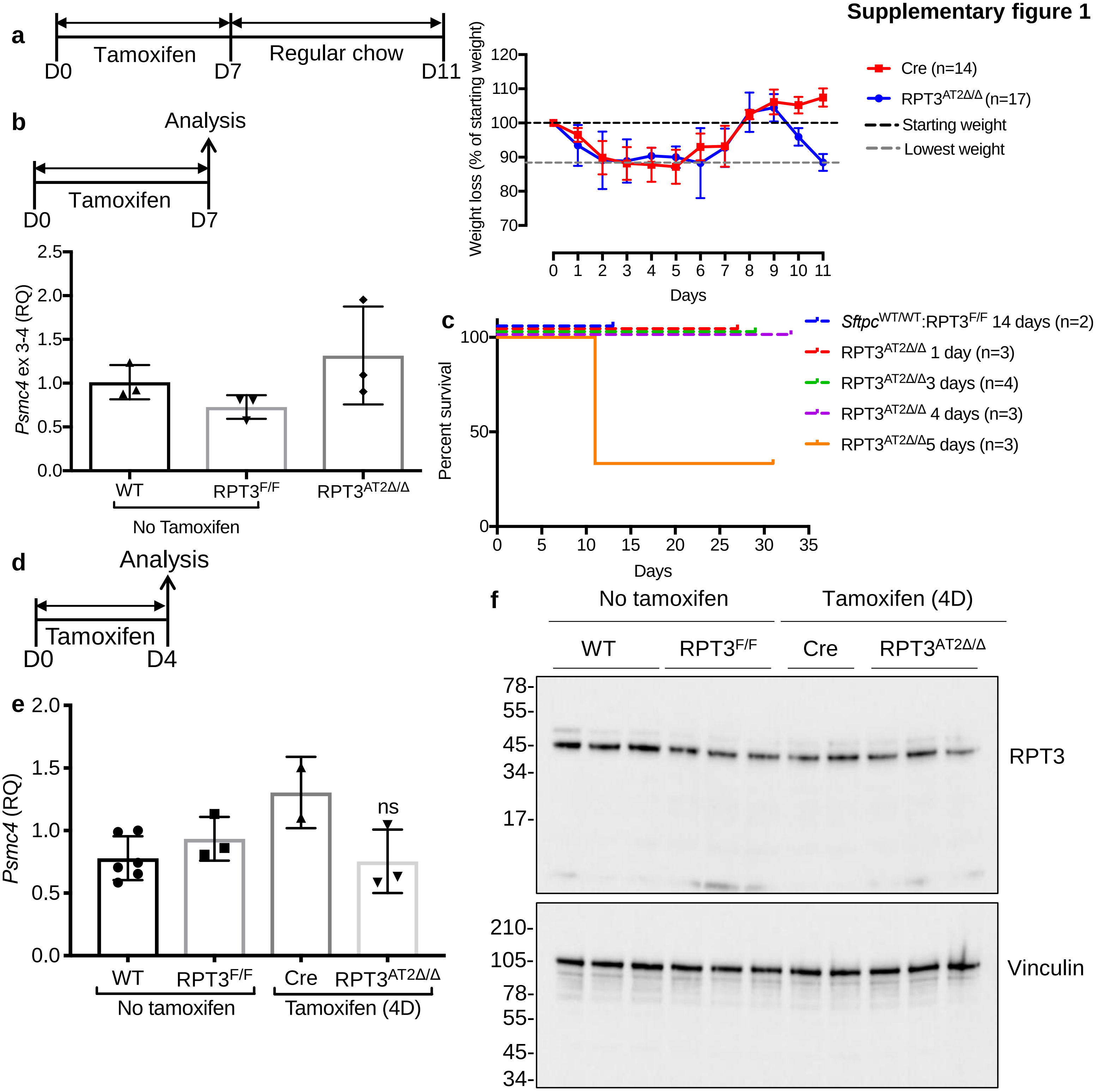

Supplementary figure 2

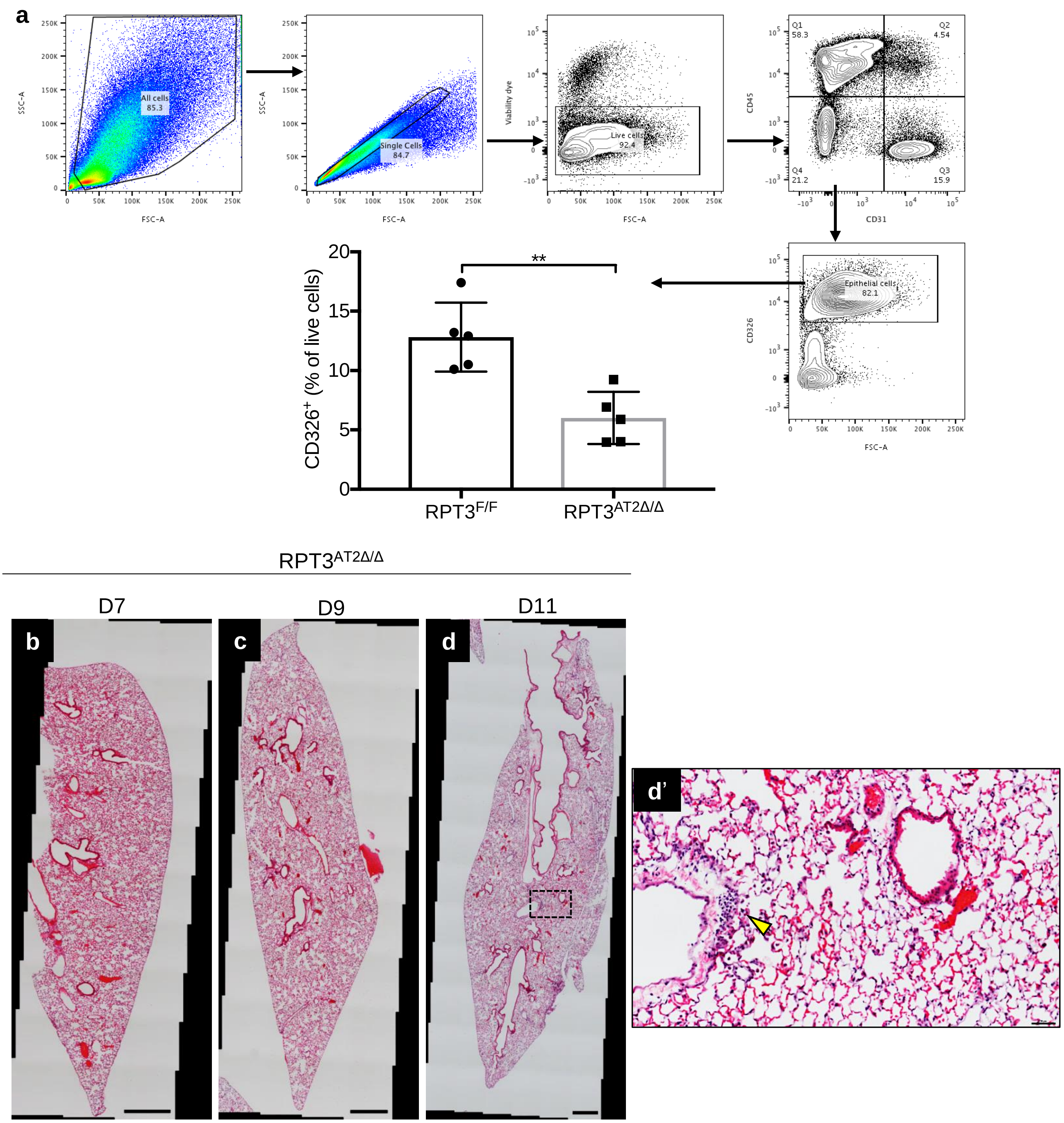

Supplementary figure 3

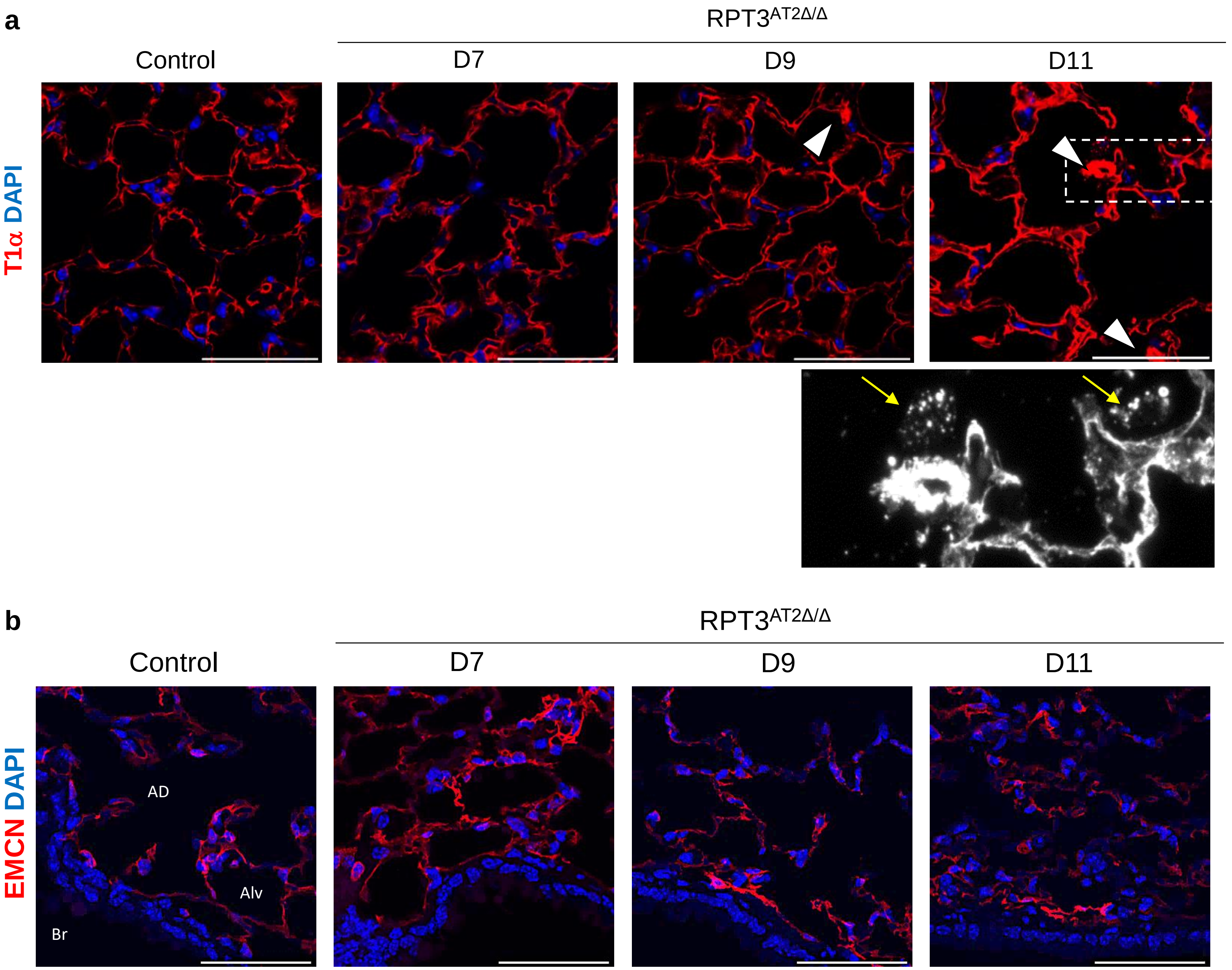

Supplementary figure 4

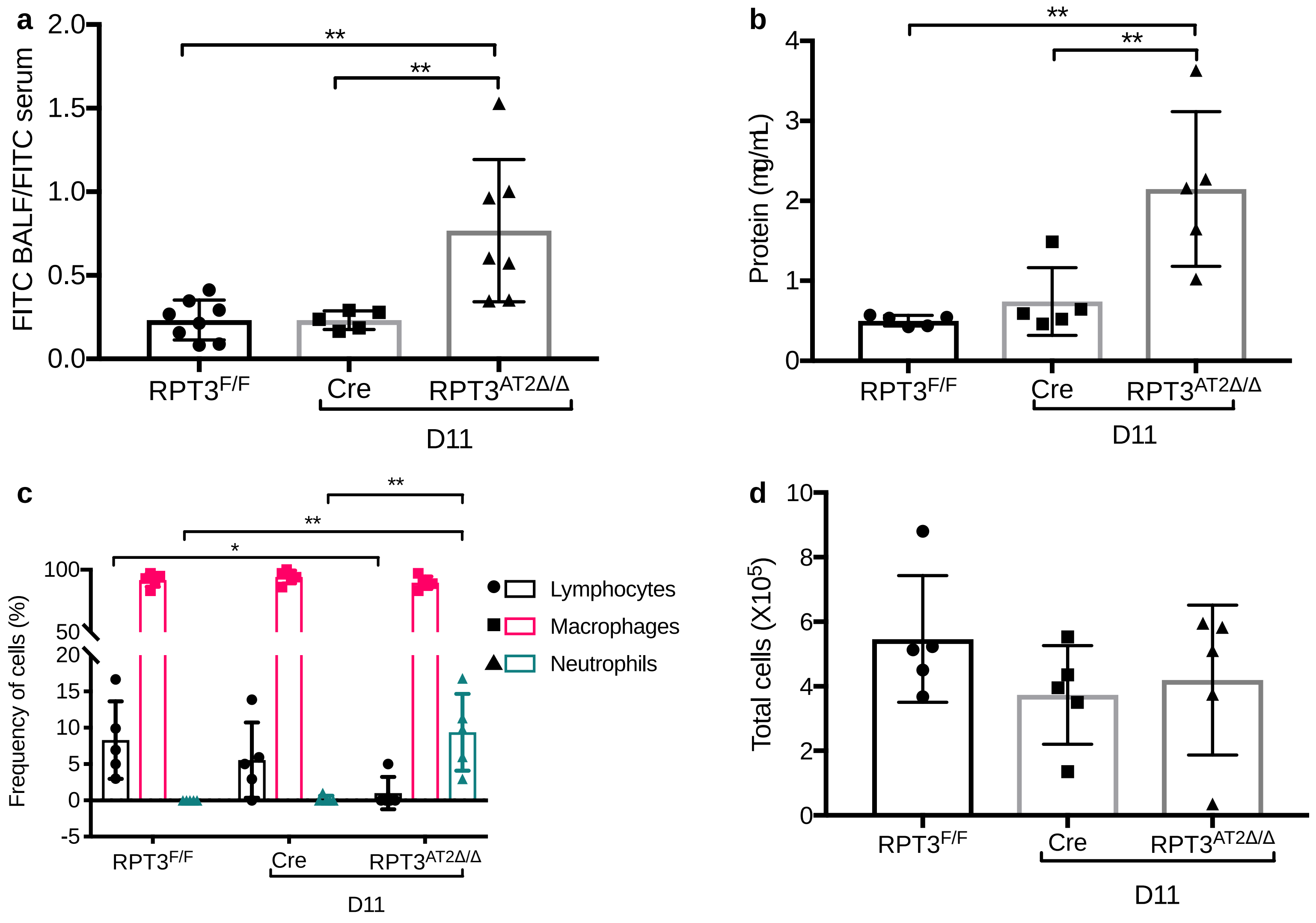

Supplementary figure 5

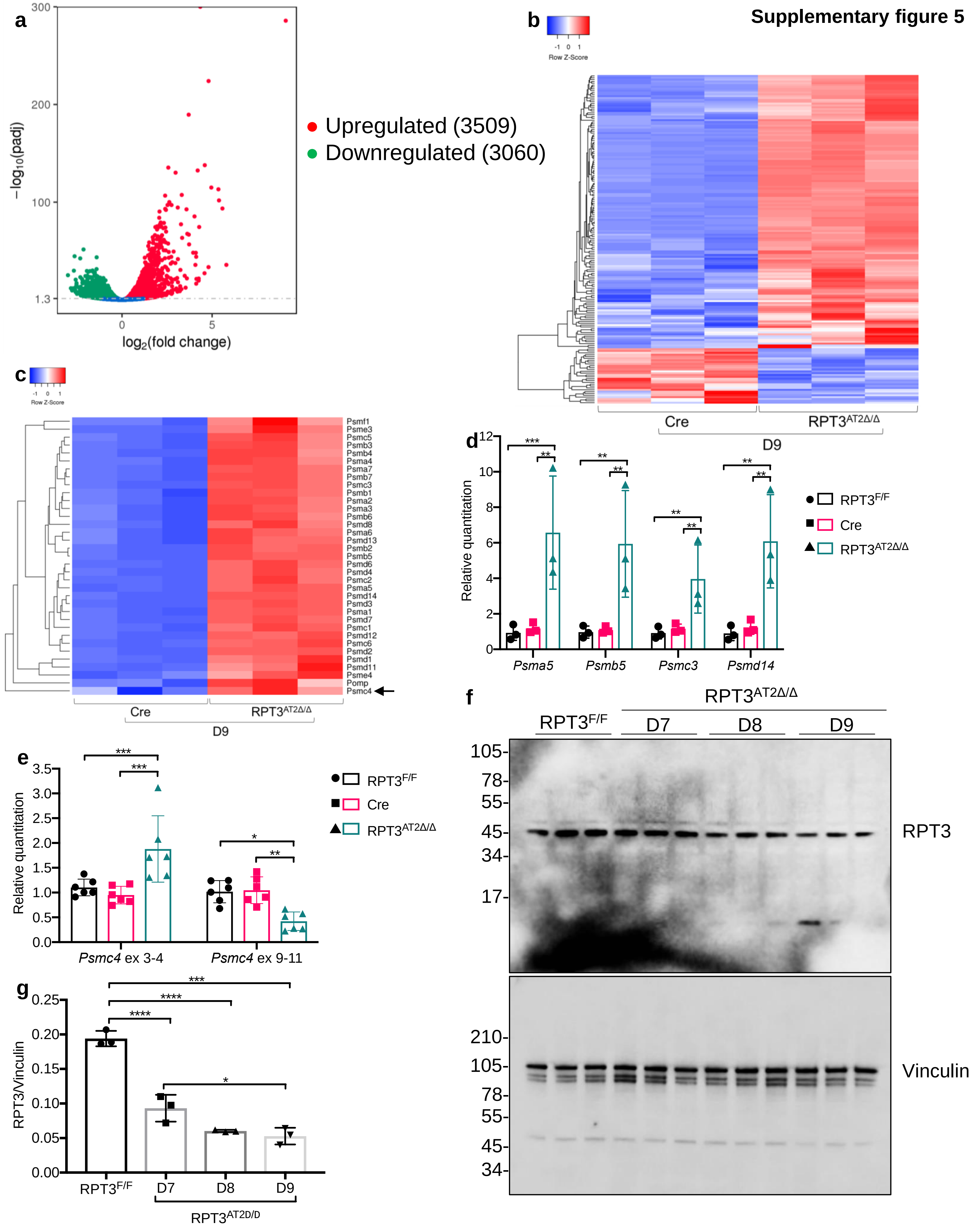

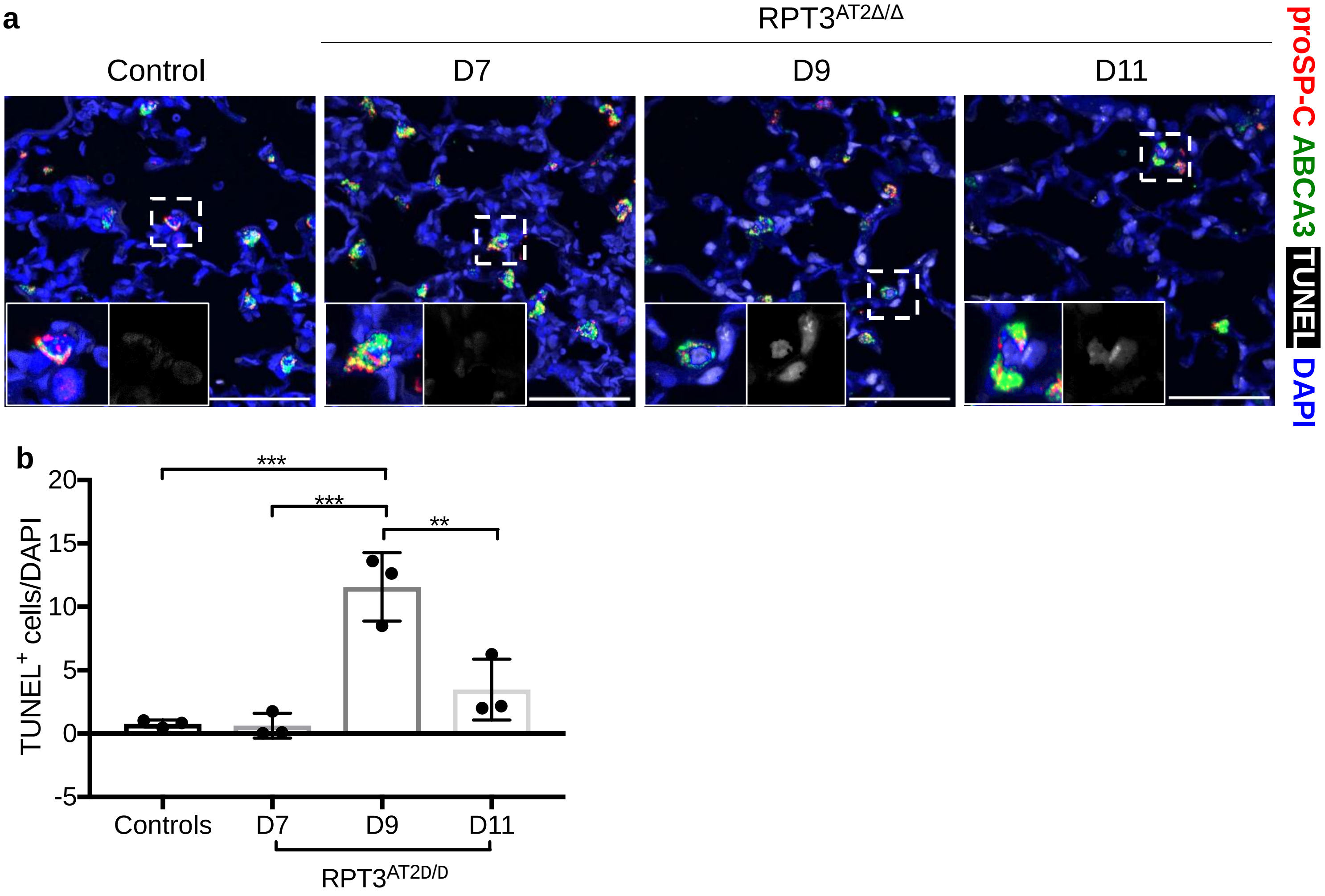
a
RPT3AT2Δ/Δ
Control
D7
D9
D11
proSP-C ABCA3 TUNEL DAPI
b
***
***
**
20
15
10
5
0
-5
TUNEL+ cells/DAPI
Controls
D7
D9
D11
RPT3AT2D/D

Supplementary figure 7

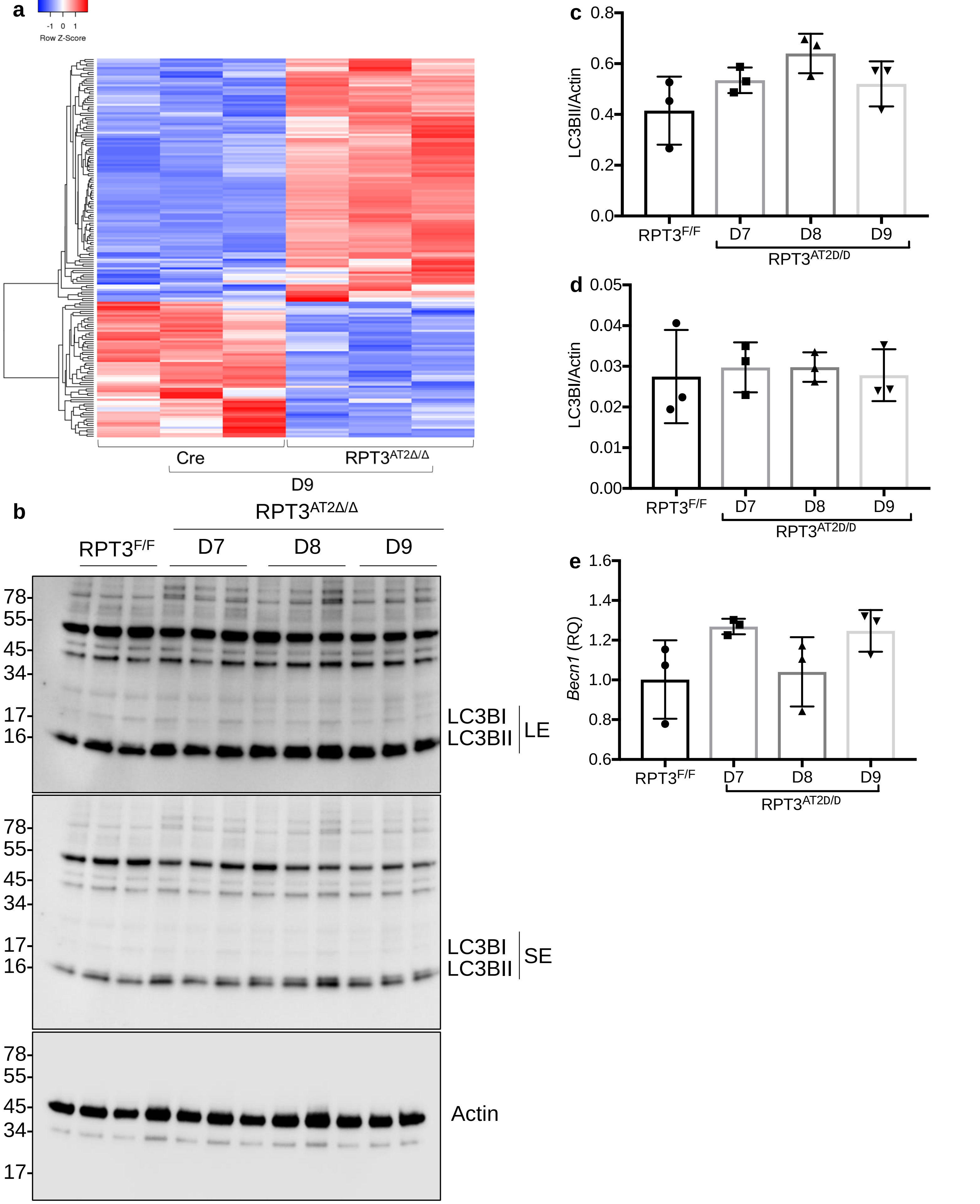

Supplementary figure 8

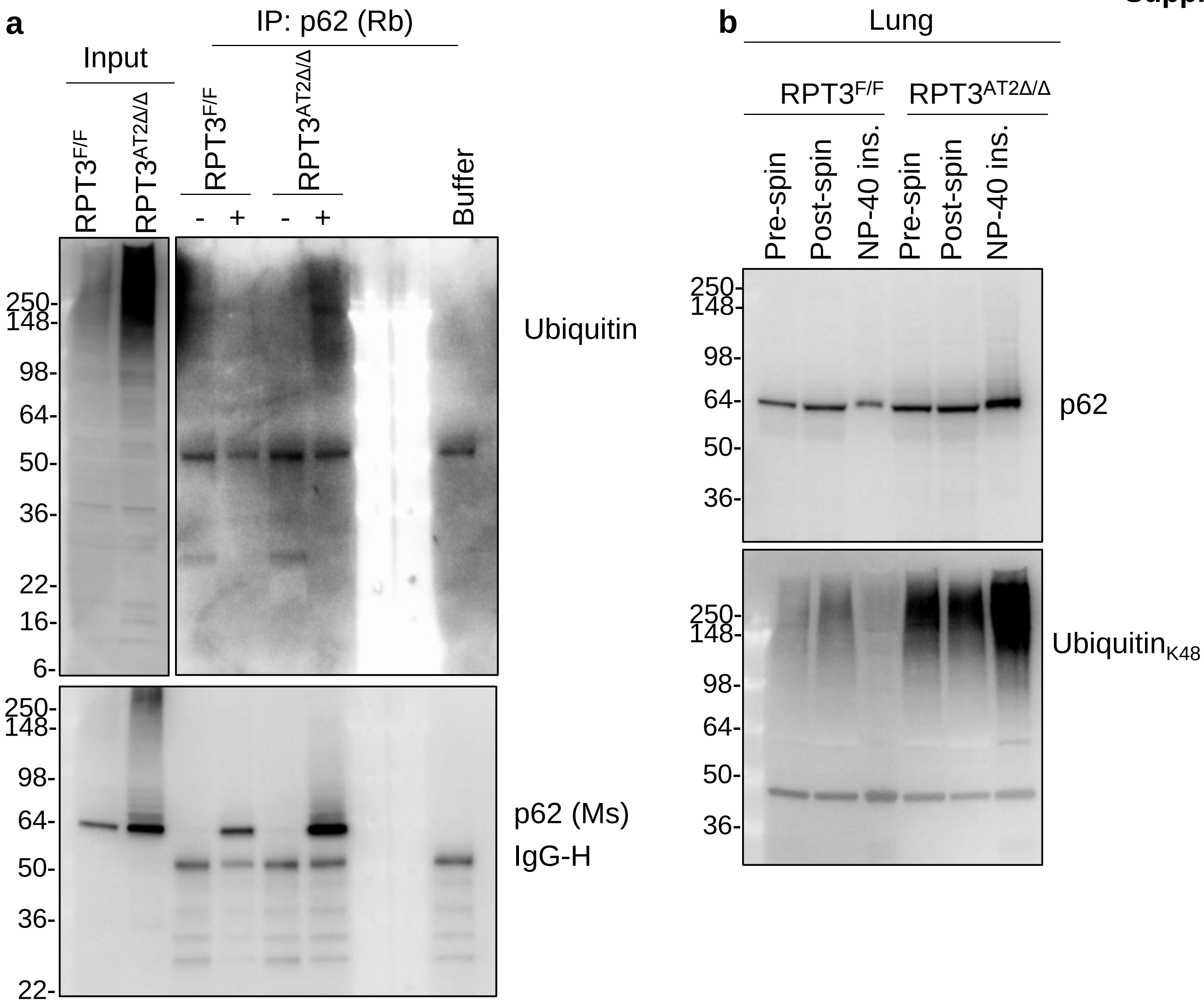

Supplementary figure 9

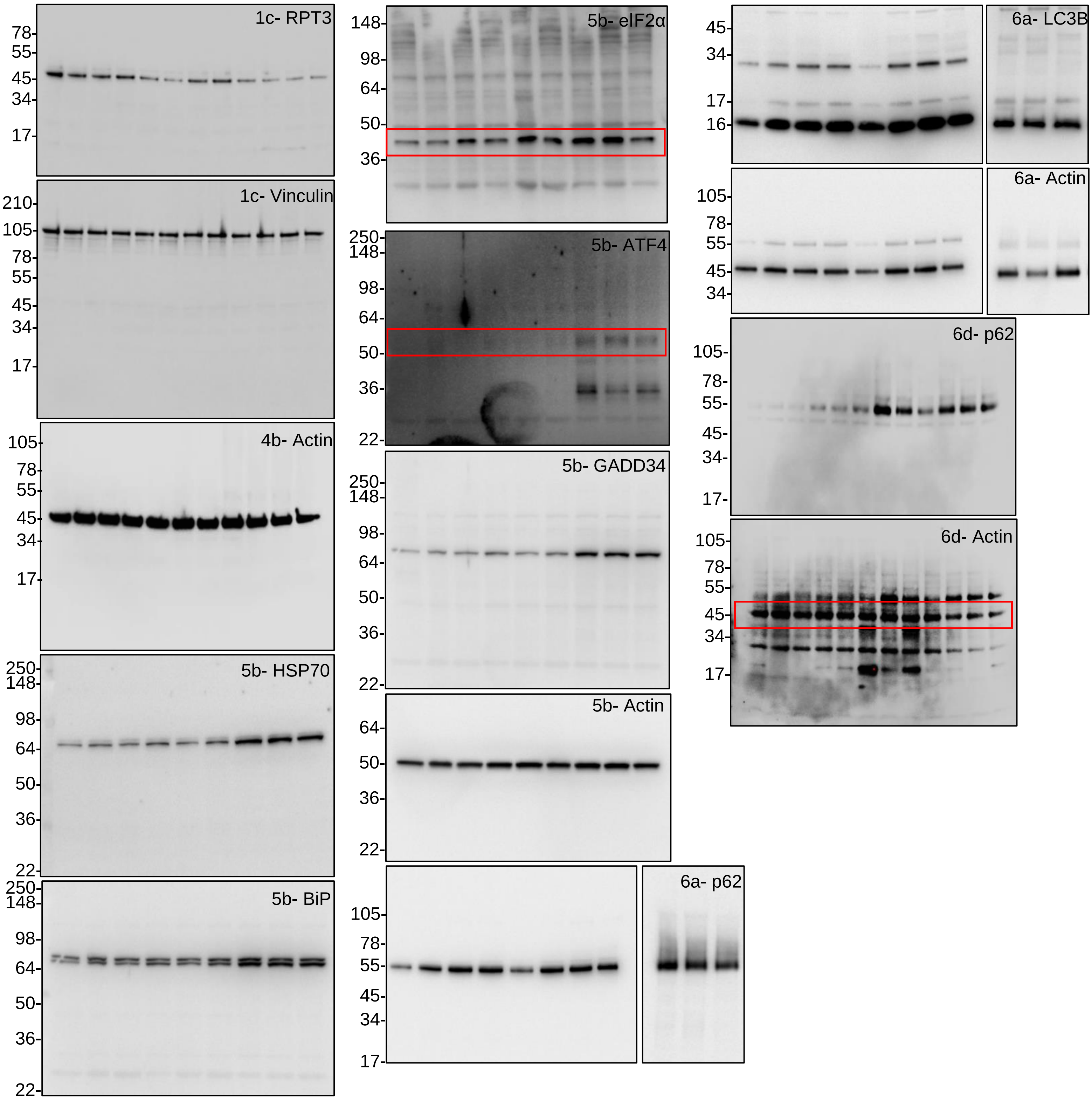

Supplementary table S1: Top 25 biological processes differentially regulated in RPT3-deficient AT2 cells isolated on D9.

ID	Name	p-value	Hit Count in Query List	Hit Count in Genome
GO:0044248	cellular catabolic process	1.35E-21	456	1828
GO:0019752	carboxylic acid metabolic process	6.36E-20	279	1003
GO:0043436	oxoacid metabolic process	2.03E-19	301	1114
GO:0031329	regulation of cellular catabolic process	2.71E-19	202	663
GO:0006082	organic acid metabolic process	3.01E-19	304	1131
GO:0009894	regulation of catabolic process	4.33E-18	224	777
GO:0022603	regulation of anatomical structure morphogenesis	1.85E-17	297	1128
GO:0006520	cellular amino acid metabolic process	1.96E-17	136	402
GO:1901575	organic substance catabolic process	2.74E-17	454	1910
GO:0012501	programmed cell death	3.65E-16	457	1952
GO:0045862	positive regulation of proteolysis	8.46E-16	127	381
GO:0080134	regulation of response to stress	8.51E-16	382	1579
GO:0080135	regulation of cellular response to stress	1.06E-15	211	752
GO:0043632	modification-dependent macromolecule catabolic process	2.00E-15	178	606
GO:0006915	apoptotic process	4.78E-15	446	1923
GO:0009057	macromolecule catabolic process	5.63E-15	307	1223
GO:0006508	proteolysis	7.52E-15	401	1697
GO:0051603	proteolysis involved in cellular protein catabolic process	1.18E-14	188	662
GO:0002479	antigen processing and presentation of exogenous peptide antigen via MHC class I, TAP-dependent	1.46E-14	38	64
GO:0019941	modification-dependent protein catabolic process	2.31E-14	173	598
GO:0010565	regulation of cellular ketone metabolic process	2.47E-14	75	188
GO:0031331	positive regulation of cellular catabolic process	3.74E-14	116	352
GO:0038061	NIK/NF-kappaB signaling	5.02E-14	55	119
GO:0006511	ubiquitin-dependent protein catabolic process	5.77E-14	170	590
GO:0060071	Wnt signaling pathway, planar cell polarity pathway	6.82E-14	53	113

Supplementary table S2: List of proteasome-associated genes upregulated in RPT3-deficient AT2 cells isolated on D9. p-values were adjusted using Benjamini and Hochberg's approach for controlling false discovery rate.

Gene name	log2 fold change	p-value (adjusted)	
Psma1	1.8291	2.41E-42	20S
Psma2	1.4393	7.14E-24	
Psma3	1.4547	6.63E-22	
Psma4	1.7351	1.25E-31	
Psma5	2.1626	3.16E-76	
Psma6	1.3734	5.85E-21	
Psma7	1.9168	2.71E-39	
Psmb1	1.3771	6.95E-19	
Psmb2	1.9844	9.08E-56	
Psmb3	1.9718	3.47E-37	
Psmb4	1.9269	2.78E-42	
Psmb5	2.1724	1.75E-65	
Psmb6	1.5288	4.6E-21	
Psmb7	2.034	4.28E-48	
Psmc1	1.8213	4.06E-37	19S
Psmc2	1.6539	9.52E-50	
Psmc3	1.7957	1.2E-44	
Psmc4	0.43458	0.0012686	
Psmc5	1.6122	5.4E-29	
Psmc6	2.1024	7.05E-78	
Psmd1	2.1492	1.53E-54	
Psmd11	2.2802	3.72E-60	
Psmd12	1.9276	2.16E-57	
Psmd13	1.6117	1.78E-24	
Psmd14	2.2899	4.52E-74	
Psmd2	1.9338	9.27E-63	
Psmd3	1.9437	6.04E-76	
Psmd4	2.1525	2.91E-60	
Psmd6	1.9964	2.91E-55	
Psmd7	1.3706	1.12E-35	
Psmd8	1.4197	9.74E-22	
Pomp	1.1466	8.13E-11	Regulators
Psme3	0.72585	3.67E-10	
Psme4	1.4567	1.18E-25	
Psmf1	0.66175	0.00000193	

Supplementary table S3: List of primary antibodies used for Western blot (WB) and immunofluorescence (IF) analyses.

Antibody	Source	Catalog number	Host	Dilution (WB)	Dilution (IF)	Antigen retrieval
ABCA3	In house	985	Guinea pig		1:100	Sodium citrate
ABCA3	Seven Hills	70565	Rabbit		1:100	Sodium citrate
Actin	Seven Hills Bioreagent	LMAB-C4	Mouse	1: 40,000		
ATF4	Cell Signaling Technologies	11815, clone D4B8	Rabbit	1:1000		
BiP	Sigma-Aldrich	G9043, clone ET-21	Rabbit	1:30,000		
eIF2α	Abcam	5369	Mouse	1:1000		
EMCN	R&D systems	AF4666	Goat		1:200	Sodium citrate
GADD34	Thermo Fisher Scientific	PA1-139	Rabbit	1:1000		
HSP70	Abcam	2787, clone 5A5	Mouse	1:1000		
K-48 Ubiquitin	Millipore Sigma	05-1307, clone Apu2	Rabbit	1: 1000		
LC3B	Novus Biologicals	NB100-2220	Rabbit	1:1000		
p62	Abcam	56416	Mouse	1: 1000	1:100	Sodium citrate
proSP-C	In house	992	Guinea pig		1:1000	Compatible with sodium citrate
proSP-C	Seven Hills	09337	Rabbit		1:1000	Compatible with sodium citrate
RPT3 (TBP7)	Abcam	139184, clone EPR9911 [B]	Rabbit	1:10,000		
RPT5	Bethyl Laboratories	A303-538A	Rabbit	1:2000		
T1α	Developmental Studies Hybridoma bank	531893	Hamster		1:100	Sodium citrate
Ubiquitin	Enzo Life Sciences	BML-PW8810-0100, clone FK2	Mouse	1:2000		
Vinculin	Sigma	V9131, clone hVIN-1	Mouse	1: 60,000		
α1–7	Abcam	22674, clone MCP231	Mouse	1:1000		

Supplementary table S4: List of secondary antibodies used for Western blot (WB) and immunofluorescence (IF) analyses.

**Antibody**	**Source**	**Catalog number**	**Dilution (WB)**	**Dilution (IF)**
Donkey anti goat Alexa 568	Invitrogen	A11057	-	1:200
Donkey anti guinea pig Alexa 488	Jackson ImmunoResearch	706-545-148		
Donkey anti guinea pig Alexa 647	Jackson ImmunoResearch	706-605-148		
Donkey anti rabbit Alexa 568	Invitrogen	A10042		
Donkey anti rabbit Alexa 647	Invitrogen	A31573		
Goat anti guinea pig Alexa 488	Invitrogen	A11073		
Goat anti guinea pig Alexa 568	Invitrogen	A11075		
Goat anti guinea pig Alexa 647	Invitrogen	A21450		
Goat anti hamster 568	Invitrogen	A21112		
Goat anti mouse Alexa 647	Invitrogen	A21236		
Goat anti rabbit Alexa 488	Invitrogen	A11034		
Goat anti rabbit Alexa 568	Invitrogen	A11011		
Goat anti mouse HRP	EMD Millipore	401215	1:20000	-
Goat anti rabbit HRP	EMD Millipore	401315		
